# Inhibition of β-site amyloid precursor protein cleaving enzyme 1 and cholinesterases by pterosins via a specific structure−activity relationship with a strong BBB permeability

**DOI:** 10.1038/s12276-019-0205-7

**Published:** 2019-02-12

**Authors:** Susoma Jannat, Anand Balupuri, Md Yousof Ali, Seong Su Hong, Chun Whan Choi, Yun-Hyeok Choi, Jin-Mo Ku, Woo Jung Kim, Jae Yoon Leem, Ju Eun Kim, Abinash Chandra Shrestha, Ha Neul Ham, Kee-Ho Lee, Dong Min Kim, Nam Sook Kang, Gil Hong Park

**Affiliations:** 1 Department of Biochemistry and Molecular Biology, College of Medicine, Korea Molecular Medicine and Nutrition Research Institute, Korea University, Seoul, 02841 Korea; 2 Graduate School of New Drug Discovery and Development, Chungnam National University, Daejeon, 34134 Korea; 3 Bio-Center, Gyeonggido Business & Science Accelerator, Suwon, 16229 Korea; 4 College of Pharmacy, Woosuk University, Wanju, Jeonbuk 55338 Korea; 5 Division of Radiation Cancer Research, Korea Institute of Radiological and Biomedical Sciences, Seoul, 01812 Korea; 6 Department of Creative Arts Psychotherapy, College of Cultural Convergence, Jeonju University, Jeonju, 55069 Korea; 7 Department of Chemistry and Biochemistry, Faculty of Arts and Science, Concordia University, 7141 Sherbrooke St. W., Montreal, QC H4B 1R6 Canada; 8 Center for Structural and Functional Genomic, Department of Biology, Faculty of Arts and Science, Concordia University, 7141 Sherbrooke St. W., Montreal, QC H4B 1R6 Canada

**Keywords:** Alzheimer's disease, Alzheimer's disease, Biologics

## Abstract

We extracted 15 pterosin derivatives from *Pteridium aquilinum* that inhibited β-site amyloid precursor protein cleaving enzyme 1 (BACE1) and cholinesterases involved in the pathogenesis of Alzheimer’s disease (AD). (2*R*)-Pterosin B inhibited BACE1, acetylcholinesterase (AChE) and butyrylcholinesterase (BChE) with an IC_50_ of 29.6, 16.2 and 48.1 µM, respectively. The *K*_i_ values and binding energies (kcal/mol) between pterosins and BACE1, AChE, and BChE corresponded to the respective IC_50_ values. (2*R*)-Pterosin B was a noncompetitive inhibitor against human BACE1 and BChE as well as a mixed-type inhibitor against AChE, binding to the active sites of the corresponding enzymes. Molecular docking simulation of mixed-type and noncompetitive inhibitors for BACE1, AChE, and BChE indicated novel binding site-directed inhibition of the enzymes by pterosins and the structure−activity relationship. (2*R*)-Pterosin B exhibited a strong BBB permeability with an effective permeability (*P*_e_) of 60.3×10^−6^ cm/s on PAMPA-BBB. (2*R*)-Pterosin B and (2*R*,3 *R*)-pteroside C significantly decreased the secretion of Aβ peptides from neuroblastoma cells that overexpressed human β-amyloid precursor protein at 500 μM. Conclusively, our study suggested that several pterosins are potential scaffolds for multitarget-directed ligands (MTDLs) for AD therapeutics.

## Alzheimer’s disease: Promising therapeutic compounds found in plants

Compounds extracted from bracken fern block the activity of three enzymes associated with Alzheimer’s disease (AD). Because AD is a complex and multifactorial disease, a multitarget-directed approach is an attractive strategy for the development of disease-modifying therapeutics. A study led by Gil Hong Park, Korea University, Seoul, and Nam Sook Kang, Chungnam National University, Daejon, revealed that pterosin derivatives could reduce the activity of β-site amyloid precursor protein cleaving enzyme 1, acetylcholinesterase and butyrylcholinesterase in a concentration-dependent manner. Furthermore, the fern-extracted compounds did not cause cellular toxicity and were able to cross the blood–brain barrier, which is impermeable to most drugs, to reach the brain. Future studies will determine whether they can be developed into drugs to simultaneously engage various AD targets in animal models of the disease.

## Introduction

Alzheimer’s disease (AD) is an age-related neurodegenerative disorder with characteristic clinical and pathological features, which are associated with the loss of neurons in certain brain areas, leading to memory impairment, cognitive dysfunction, behavioral disturbances, deficits in activities of daily living, and eventually death^1,2^. The symptoms of AD include dementia, apraxia, aphasia, depression, a short attention span, visuospatial navigation deficits, anxiety, and delusions. AD affects up to 5% of individuals aged above 65 years and increases to 20% in those above 80 years of age. Approximately 46.8 million individuals suffer from AD worldwide, accounting for an estimated annual societal economic cost of $818 billion in 2015, which is expected to increase to $1 trillion in 2018 and $2 trillion in 2030^3^.

Reflecting the multifactorial and complex etiology of AD, the histopathological hallmarks, such as amyloid β-protein (Aβ) deposits^4^; dysfunctional signaling of acetylcholine (ACh) in certain areas of the brain^5^; τ protein neurofibrillary tangles^6^; metabolic pathways, such as those involving cAMP-responsive element binding protein (CREB)^7^; oxidative stress^8^; and inflammation^9^, appear to play significant roles. The two most common hypotheses include amyloid and cholinergic hypotheses.

The amyloid hypothesis suggests that the accumulation and oligomerization of Aβ peptide in the brain plays a critical role in AD pathogenesis^10^. It has been clearly established that the overproduction of Aβ by the aspartic protease BACE1 and subsequent oligomerization result in toxic amyloid oligomers inducing neurodegeneration. BACE1 cleaves β-amyloid precursor protein (APP) and forms approximately 90% of the Aβ peptides^11,12^. Endogenous BACE1 activity is increased in the brain of patients with sporadic AD^13^. In addition, emerging evidence shows significant elevation of BACE1 in the presence of other AD risk factors, such as traumatic brain injury, stroke, and cardiovascular events, which suggests that BACE1 is a stress-response protein and its activities increase during AD risk factor-related events^14^. However, all previously discovered natural and synthetic BACE1 inhibitors have failed clinical trials as therapeutic candidates for AD mainly due to adverse effects, such as ocular toxicity^15^ and poor BBB penetration with low brain:plasma concentration ratios^16^. Currently, no BACE1 inhibitors have been approved for the clinical treatment of AD.

The cholinergic hypothesis proposes a massive loss of cholinergic neurons as a downstream phenotypic consequence in the pathogenesis of AD^1^. A decreased level of ACh, a neurotransmitter, in the brain plays a critical role in the progression of AD. ACh plays an important role in the cognitive mechanism and is hydrolyzed by AChE and BChE, leading to the loss of cognitive functions. With the progression of AD, the activity of AChE decreases, while that of BChE increases to compensate for the loss of AChE in an attempt to modulate ACh levels in cholinergic neurons and augment learning^17,18^. Both enzymes are therapeutic targets to combat cognitive deficits at different stages of AD with AChE in the early stage and BChE in the later stages^19^. In particular, AChE inhibitors, such as E2020 (donepezil), have become the drug of choice in the clinical management of AD. However, E2020 lacks BACE1-inhibitory activity and fails to provide pathogenetic treatment along with various adverse side effects^20^.

After several decades of research efforts, the treatment of AD continues to face a significant unmet need, with therapies based largely on the cholinesterase inhibitors rivastigmine, E2020, and galantamine, with the only exception an NMDA (*N*-methyl-d-aspartate) receptor antagonist, memantine^21^. However, these drugs only relieve AD symptoms for a short period of time without reversing disease progression. In recent years, the need for disease-modifying drugs for AD has been addressed via new approaches to design structures with different targets involved in the pathogenesis of AD, including multitarget-directed ligands (MTDLs), in line with the multifactorial and complex etiology of AD to produce the desired therapeutic efficacy^22,23^. Thus, MTDLs, such as dual- and multiacting anti-AD agents, represent a promising strategy for the treatment of AD, engaging different targets simultaneously as a hybrid.

Ferns belong to the botanical group Pteridophyta. Several ferns have been used in ethnopharmacy to treat various illnesses^24^. Alkaloids present in *Huperzia serrata*, particularly huperzine A, B, and R and 8-β phlegmariurine B, have been used for AD therapy as cholinesterase inhibitors^25^. *Pteridium aquilinum* (*P. aquilinum*) is distributed globally and has been widely consumed traditionally as foods in Korea and Japan. Pterosins, the major components of *P. aquilinum*, have been reported to be nontoxic to humans, although some of its components, such as the unstable glucoside ptaquiloside, are carcinogenic^26,27^. Recently, pterosin B was shown to prevent chondrocyte hypertrophy and osteoarthritis in mice at least in part via CREB activation^28^. In addition, pterosin derivatives, particularly pterosin A, showed pharmacological properties relevant to the prevention and treatment of diabetes and obesity in mice^29,30^. However, the anti-AD activity of pterosins has not been reported to date.

The present study was performed to characterize the anti-AD potential of pterosin derivatives by investigating their activities in vitro to inhibit BACE1, AChE and BChE as well as BBB permeability. In addition, the structure−activity relationship (SAR) was analyzed using molecular docking simulations to determine the molecular interactions between pterosins and the active sites of BACE1, AChE and BChE.

## Materials and methods

### Chemicals

BACE1 (human recombinant β-secretase) (EC 3.1.1.8) and the BACE1 FRET (fluorescence resonance energy transfer) assay kit Red were purchased from ThermoFisher Scientific (P2985; Waltham, MA, USA). Electric eel AChE (EC3.1.1.7), horse serum BChE (EC 3.1.1.8), acetyl thiocholine iodide, butyryl thiocholine chloride, 5,5′-dithiobis(2-nitrobenzoic acid) (DTNB), quercetin, berberine, and the MTT assay kit were purchased from Sigma-Aldrich Co. (St. Louis, MO, USA). For the cell culture experiment, Dulbecco’s modified Eagle’s medium (DMEM), Opti-MEM, fetal bovine serum (FBS), and penicillin/streptomycin were obtained from Capricorn Scientific (Ebsdorfergrund, Germany). β-secretase inhibitor III (β-SI) was purchased from Calbiochem (Darmstadt, Germany). The protease inhibitor mixture (a mixture of AEBSF, pepstatin A, E-64, bestain, leupeptin, and aprotinin) was purchased from Sigma-Aldrich (St. Louis, MO, USA). Enzyme-linked immunosorbent assay (ELISA) kits for Aβ40 and Aβ42 were obtained from IBL (Kunma, Japan). All chemicals and solvents used were reagent grade, purchased from commercial sources, and used as received.

### Plant material

*P. aquilinum* was collected from the mountainous regions of Gapyeong-gun, Gyeonggido, Korea, and authenticated by Prof. Ki-Joong Kim (Korea University, Seoul). A voucher specimen was deposited in the laboratory of Prof. Gil Hong Park.

### Extraction, fractionation, and identification of pterosin derivatives from *P. aquilinum*

**Fig. 1:**
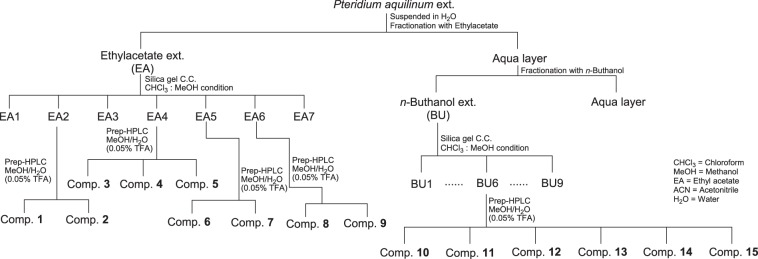
Flow chart to illustrate the process to isolate and purify pterosin derivatives from *Pteridium aquilinum*

Pterosin derivatives were isolated from *P. aquilinum* using hot water followed by various chromatographic methods (Fig. 1). The hot water extract obtained by refluxing 250 g of the whole plants of *P. aquilinum* in 1.5 L H_2_O for 24 h in a steamer (OSK-2002, Red Ginseng Doctor, Well sosanaTM, Daewoong Pharmaceutical, Seoul, Korea) was initially partitioned with an equal volume of EtOAc and subsequently with *n*-BuOH. The repeated column chromatographic separation of the EtOAc-soluble fraction and the *n*-BuOH-soluble fraction resulted in the isolation of 15 pterosin derivatives with purities greater than 97%. The structures of the isolated compounds were identified via analysis of the spectral data, including MS, 1D- and 2D-NMR, as well as comparison of their data with the published values, including heteronuclear single quantum correlation and heteronuclear multiple bond correlation. The pterosin compounds were dissolved in Dimethyl Sulfoxide (DMSO) for use in the experiment.

### In vitro BACE1 enzyme assay

The BACE1 FRET assay was carried out according to the manufacturer’s instructions with slight modifications as previously described^31^. The assay was performed in a 381-well black plate using a multi-pipette. Readings were obtained two times, the first time at 0 min and the second time after 60 min incubation at room temperature (26 °C) and stopping the reaction with BACE1 stop buffer, using a spectrofluorometer (Gemini EM; Molecular Devices, San Jose, CA, USA) at 545 nm (excitation) and 590 nm (emission). Quercetin was used as the positive control. % Inhibition calculation: [1 – (60 min value - 0 min value/control)] × 100.

### In vitro cholinesterase enzyme assay

The inhibitory activities of pterosin derivatives against cholinesterases were measured using the method developed by Ellman et al.^32^. The reaction mixture contained 140 μL of sodium phosphate buffer (pH 8.0), 20 μL of test sample solution (final concentration of the compound 100 μM), and 20 μL of AChE or BChE solution, mixed and incubated for 15 min at room temperature. All test samples and the positive control (berberine) were diluted or dissolved in 10% analytical grade ethanol. The reactions were initiated following the addition of 10 μL of DTNB and 10 μL of acetyl thiocholine iodide for the AChE assay or butyryl thiocholine chloride for the BChE assay in 96-well microplates, which were incubated for 15 min. The hydrolysis of acetyl thiocholine iodide or butyryl thiocholine chloride was monitored by tracking the formation of yellow 5-thio-2-nitrobenzoate anion resulting from the reaction between DTNB and thiocholine released by the enzyme, using a microplate spectrophotometer (Molecular Devices, Sunnyvale, CA, USA) at 412 nm. The percent inhibition was calculated as (1−*S/E*) × 100, where *S* and *E* represent the enzyme activities with and without the test sample, respectively.

### Kinetic parameters of BACE1, AChE and BChE inhibition by pterosin derivatives and the inhibition mechanism

To determine the *K*_i_ and the mode of enzymatic inhibition of the most active compounds against BACE1, AChE, and BChE, Dixon and Lineweaver−Burk plots were employed. The *K*_i_ was determined by interpretation of the Dixon plot, with the value of the *x*-axis intercept taken as *−K*_i_. The Dixon plot is a graphical method [plot of 1/enzyme velocity (1/*V*) against inhibitor concentration (*I*)] for the determination of *K*_i_ and the type of enzyme inhibition for the enzyme–inhibitor complex. Dixon plots for enzyme inhibition by pterosins were tested in the presence of different substrate concentrations: 150, 250, and 750 nM for BACE1, 100, 300, and 600 µM for AChE, and 200, 400, and 800 µM for BChE. Lineweaver−Burk plots were analyzed in the presence of different inhibitor concentrations: 0–125 µM for BACE1, 0–100 µM for AChE, and 0–150 µM for BChE.

### Molecular docking

Docking studies were performed on BACE1, AChE, and BChE to understand the inhibition profile of the pterosin derivatives. The X-ray crystal structures of human BACE1 complexed with 2-amino-3-{(1r)-1-cyclohexyl-2-[(cyclohexylcarbonyl)amino]ethyl}-6-phenoxyquinazolin-3-ium (QUD) (PDB code: 2WJO)^33^, human AChE complexed with E2020 (PDB code: 4EY7)^34^ and human BChE complexed with *N*-{[(3R)-1-(2,3-dihydro-1H-inden-2-yl)piperidin-3-yl]methyl}-*N*-(2-methoxyethyl)naphthalene-2-carboxamide (3F9) (PDB code: 4TPK)^35^ were retrieved from the RCSB Protein Databank (PDB, https://www.rcsb.org/). Discovery Studio 2017 R2 (BIOVIA, San Diego: Dassault Systèmes) was used to create 3D structures of the docked ligands and for energy minimization. Docking studies were carried out using AutoDock 4.2.6 software^36^. Protein structures were held rigid, whereas ligands were treated as fully flexible during docking. Prior to docking, the protein and ligand structures were processed with AutoDock Tools (ADT) 1.5.6^37^. The cocrystallized ligands and water molecules were removed from the original PDBs. Polar hydrogen atoms were merged, and Kollman and Gasteiger charges were assigned to the protein structures. Gasteiger charges were added by default to the ligands for docking calculations. The number of rotatable bonds was set, and all torsions were allowed to rotate. For each enzyme, the AutoGrid program was employed to create a grid box of size 60 × 60 × 60 Å^3^ with 0.375 Å spacing. The center of the grid was defined according to a recent study, which reported different sites for catalytic, mixed-type and noncompetitive BACE1, AChE, and BChE inhibitors^38^. Lamarckian genetic algorithm was used for the conformational search^39^. The docking protocol consisted of 100 runs, 25×10^5^ energy evaluations and 27,000 iterations. Other docking parameters were set to the default values. Docked poses were selected on the basis of scoring functions and protein−ligand interactions. Binding interaction figures were generated using Discovery Studio 2017 R2.

### PAMPA-BBB procedure

PAMPA (parallel artificial membrane permeation assay) was used as a high-throughput assay to predict BBB permeation^40^. Porcine polar brain lipid (PBL) was used as an artificial membrane to predict BBB permeation. Initially, the test compound was dissolved in DMSO at 5 mg/mL. This compound stock solution (10 µL) was diluted 200-fold in a universal buffer at pH 7.4 and mixed with a multi 8-channel pipette to obtain the secondary stock solution (final concentration 25 µg/mL). The secondary stock solution (200 µL) was placed in the donor wells. The filter membrane was coated with PBL in dodecane (4 mL volume of 20 mg/mL PBL in dodecane), and 5 µL of BBB-1 lipid solution was spread on PBL by pipette. The acceptor well was filled with 200 mL of Acceptor Sink Buffer. The acceptor filter plate was cautiously positioned on the donor plate to assemble a “sandwich” (comprising an aqueous acceptor, artificial lipid membrane and aqueous donor with test compound on the top, middle and bottom, respectively). The test compound diffuses from the donor well to the acceptor well through the lipid membrane. After 4 h of incubation at pH 7.4 and 25 °C, the concentration of the compound in the acceptor, donor, reference and blank wells was estimated with a UV plate reader, Epoch Microplate Spectrophotometer (BioTek Instruments, Inc., Winooski, VT, USA). The effective permeability (*P*_e_) of the compounds was calculated using Pion PAMPA Explorer software. Samples were analyzed in quadruplets, and the average of the four runs was reported. To monitor the consistency of the analysis set, quality control standards were run with each sample set. Verapamil was employed as a high permeability standard (*P*_e_ = 16 × 10^−6^ cm/s).

### Cell culture and treatment

Mouse neuroblastoma N2a cells stably overexpressing the human APP Swedish mutation (APPswe) were kindly provided by Dr. Takeshi Iwatsubo (The University of Tokyo). The cells were cultured in 45% DMEM, 55% opti-MEM, 10% FBS, 1% penicillin-streptomycin, 1% glutamine and 0.09% hygromycin B (Sigma-Aldrich, St. Louis, MO, USA) in a humidified atmosphere with 5% CO_2_ at 37 °C. To analyze the effect of (2*R*)-pterosin B or (2*R*,3*R*)-pteroside C on APP metabolites, APPswe-containing neuroblastoma cells were cultured up to confluency in DMEM that contained 10 mM butyric acid to drive protein expression in the presence of 12, 60, 250, and 500 μM of (2*R*)-pterosin B or (2*R*,3*R*)-pteroside C for 24 h. β-SI (10 µM) was used as a positive control. The negative control included cells cultured in the absence of test compounds. Cells were lysed in immunoprecipitation buffer (50 mM Tris, pH 7.4, 150 mM NaCl, 5 mM Ethylenediaminetetraacetic Acid (EDTA), 0.5% Nonidet P-40, and 0.5% sodium deoxycholate) supplemented with the protease inhibitor mixture. Conditioned medium was collected in 0.5 mM phenylmethylsulfonyl fluoride and subjected to ELISA for the 40-residue peptide Aβ (1–40) (Aβ40) or the 42-residue Aβ (1–42) (Aβ42).

### Aβ ELISA

Aβ40 and Aβ42 ELISA was performed according to the manufacturer’s instructions. The assay was conducted in a microplate coated with anti-human Aβ_35-40_ mouse IgG (1A10) or anti-human Aβ_38-42_ mouse IgG (44A3), respectively. The microplate was added with 100 μL conditioned media, incubated overnight at 4 °C, and then washed with washing buffer. Horseradish peroxidase-conjugated anti-human Aβ_11-28_ mouse IgG (82E1) was added into each well, incubated at 4 °C for 1 h, and washed with washing buffer. 3,3′,5,5′-Tetramethylbenzidine as a chromogenic substrate was added to each well and incubated for 30 min. The reaction was stopped by the addition of 100 μL stop solution, and the absorbance at 450 nm was measured using a model 680 microplate reader (Bio-Rad, Hercules, CA).

### 3-(4,5-Dimethylthiazol-2-yl)-2,5-diphenyltetrazolium bromide (MTT) assay

Cytotoxicity was assessed using the MTT assay^41^. Briefly, the cells were seeded into a 96-well plate at a density of 1×10^5^ cells per well in 100 µL of corresponding media and incubated at 37 °C in an incubator under 5% CO_2_ tension for 24 h. The culture media were then replaced with 100 µL of fresh serum-free media in the presence of varied concentrations of pterosin derivatives, and cells were incubated for an additional 24 h. The control was treated with an equal amount of DMSO present in the assay for a 5 mM concentration. Then, 100 µL of MTT solution (0.5 mg/mL in Phosphate Buffered Saline (PBS)) was added to each culture. To measure the proportion of surviving cells, the media were replaced with 100 µL of DMSO 2 h after the administration of MTT solution. The absorbance was measured at 570 nm using a spectrophotometric plate reader (Molecular Devices, Sunnyvale, CA, USA).

### Statistical analysis

All results were presented as the mean ± standard deviation (SD) of triplicate or quadruple experiments. Statistical evaluation was performed by one-way analysis of variance (ANOVA). The analysis was performed using Graph Pad Prism 5.01 (Graph Pad Software, Inc., La Jolla, CA, USA). *P* < 0.05 was considered statistically significant.

## Results

### Isolation and characterization of pterosin derivatives from *P. aquilinum*

**Fig. 2:**
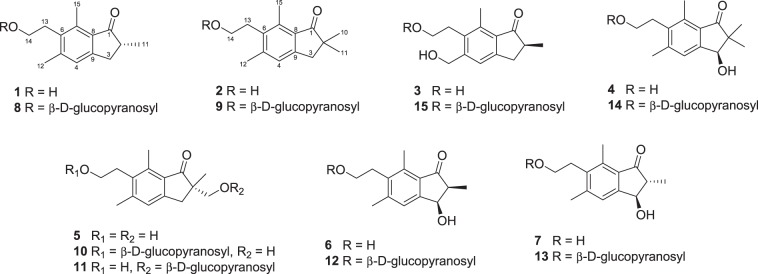
Structures of pterosin compounds **1**–**15**xxx

Pterosin derivatives were isolated from the whole plants of *P. aquilinum*. For the investigation of the phytochemical constituents from the bracken fern, a water extract was successively partitioned with ethyl acetate (EtOAc) and *n*-butanol (*n*-BuOH) (Fig. 1). Repeated column chromatography of the EtOAc-soluble fraction resulted in the isolation of nine derivatives, including (2*R*)-pterosin B (1), pterosin Z (2), (2*S*)-pterosin P (3), (3*R*)-pterosin D (4), (2*S*)-pterosin A (5), (2*S*,3*R*)-pterosin C (6), (2*R*,3*R*)-pterosin C (7), (2*R*)-pteroside B (8), and pteroside Z (9), with purities greater than 97% (Fig. 2). The repeated column chromatographic separation of the *n*-BuOH-soluble fraction resulted in the isolation of six derivatives, including (2*S*)-pteroside A (10), (2*S*)-pteroside A_2_ (11), (2*S*,3*R*)-pteroside C (12), (2*R*,3*R*)-pteroside C (13), (3*S*)-pteroside D (14), and (2*S*)-pteroside P (15), with purities greater than 97%. The structures of the compounds were identified by the analysis of spectral data, including MS, 1D- and 2D-NMR (Supplementary Information 1).

### Inhibitory activity of pterosin derivatives against BACE1, AChE, and BChE

To evaluate the anti-AD potential, the inhibitory activity of each pterosin compound against BACE1 and cholinesterases was evaluated by respective in vitro inhibition assays (Table 1). All tested pterosin derivatives showed concentration-dependent inhibitory activities against BACE1 with a range of IC_50_ values (half-maximum inhibitory concentration) of 9.74–94.4 μM, with the exception of (2*S*)-pterosin A and (2*S*)-pteroside P that were inactive at the concentrations tested, compared with the IC_50_ of quercetin used as the positive control, which was 18.8 μM. The inhibitory potency of the strongest inhibitors was in the order of (2*R*,3*R*)-pteroside C, (3*S*)-pteroside D, (2*R*)-pteroside B, (2*S*,3*R*)-pterosin C, (2*R*,3*R*)-pterosin C, (2*S*,3*R*)-pteroside C, and (2*R*)-pterosin B with IC_50_ values of 9.74, 10.7, 18.0, 23.1, 26.2, 28.9, and 29.6 μM, respectively. We subsequently tested the inhibitory potentials of the pterosin derivatives against AChE. All the tested compounds showed significant AChE-inhibitory activities, with IC_50_ values in the range of 2.55–110 μM, compared with the IC_50_ against AChE of berberine used as the positive control, which was 0.39 μM. The pterosin compounds that displayed the strongest inhibitory activity against AChE were (2*R*)-pteroside B, (2*R*,3*R*)-pteroside C, (2*S*,3*R*)-pteroside C, (2*S*,3*R*)-pterosin C, and (2*R*)-pterosin B with IC_50_ values of 2.55, 3.77, 9.17, 12.8, and 16.2 μM, respectively. Finally, we tested the inhibitory capacities of the pterosin derivatives against BChE. All the pterosin compounds tested showed inhibitory activity against BChE, with IC_50_ values that ranged from 5.29 to 119 μM, with the exception of (3*R*)-pterosin D that was inactive at the concentrations tested, compared with the IC_50_ of berberine against BChE, which was 3.32 μM. The pterosin compounds that displayed the strongest inhibitory activity against BChE were (2*R*,3*R*)-pteroside C and pteroside Z with IC_50_ values of 5.29 and 5.31 μM, respectively.

**Table 1:** IC_50_ of pterosin derivatives against BACE1, AChE, and BChE

Compounds	IC_50_ (µM)		
	BACE1	AChE	BChE
(2*S*)-Pterosin A	>125	56.7 ± 2.6	67.3 ± 3.3
(2*R*)-Pterosin B	29.6 ± 3.5	16.2 ± 1.0	48.1 ± 0.59
(2*S*,3*R*)-Pterosin C	23.1 ± 2.9	12.8 ± 0.79	44.3 ± 1.0
(2*R*,3*R*)-Pterosin C	26.2 ± 6.3	23.2 ± 4.6	20.3 ± 0.88
(3*R*)-Pterosin D	92.5 ± 7.0	68.7 ± 3.7	>125
(2*S*)-Pterosin P	67.1 ± 7.7	17.8 ± 0.62	55.9 ± 5.6
Pterosin Z	80.0 ± 5.9	46.5 ± 3.4	80.1 ± 6.8
(2*S*)-Pteroside A	84.6 ± 6.0	110 ± 3.0	19.4 ± 0.22
(2*S*)-Pteroside A_2_	94.4 ± 4.5	39.3 ± 1.9	119 ± 2.5
(2*R*)-Pteroside B	18.0 ± 2.8	2.55 ± 0.23	62.0 ± 0.71
(2*S*,3*R*)-Pteroside C	28.9 ± 2.2	9.17 ± 0.82	13.0 ± 0.14
(2*R*,3*R*)-Pteroside C	9.74 ± 1.9	3.77 ± 0.38	5.29 ± 0.82
(3*S*)-Pteroside D	10.7 ± 1.5	27.4 ± 1.2	19.3 ± 0.17
(2 )-Pteroside P	>125	57.5 ± 3.2	33.2 ± 3.0
Pteroside Z	53.3 ± 1.2	24.1 ± 1.1	5.31 ± 0.19
Quercetin^a^	18.8 ± 1.0		
Berberine^b^		0.39 ± 0.01	3.32 ± 0.12

IC_50_ values (µM) are expressed as the mean ± S.D. of three experiments

*BACE1* β-site amyloid precursor protein cleaving enzyme 1, *AChE* acetylcholinesterase, *BChE* butyrylcholinesterase

^a^Quercetin and ^b^berberine were used as positive controls for the BACE1, AChE, and BChE assays, respectively

Collectively, most of the pterosin derivatives tested exhibited significant inhibitory activities against BACE1, AChE, and BChE simultaneously. The presence of the additional 2-hydroxymethyl-tetrahydro-pyran-3,4,5-triol group as in pteroside derivatives significantly increased the inhibitory activities against the enzymes. Moreover, the presence of the additional hydroxymethyl group at position-2 of the indanone ring of (2*R*)-pterosin B as in (2*S*)-pterosin A or the methyl group as in (3*R*)-pterosin D and pterosin Z decreased the inhibitory activities against the enzymes. In particular, the presence of the hydroxymethyl group at position-5 of the indanone ring as in (2*S*)-pterosin P decreased the inhibitory activity against BACE1.

### Kinetic parameters of enzyme inhibition by pterosin derivatives

In an attempt to explain the mode of enzymatic inhibition of pterosin derivatives, we performed a kinetic analysis of BACE1 and cholinesterases for representative inhibitors (Table 2, Supplementary Information 2). A low *K*_i_ (inhibition constant) indicates tighter enzyme binding and a more effective inhibitor. Overall, the *K*_i_ values of the compounds correlated with the respective IC_50_ values. BACE1 inhibition by the compounds (2*R*,3*R*)-pteroside C, (3*S*)-pteroside D, and (2*R*,3*R*)-pterosin C was mixed-type with *K*_i_ values of 12.6, 16.5, and 27.6 µM, respectively, while inhibition by (2*R*)-pteroside B, (2*S*,3*R*)-pterosin C, and (2*R*)-pterosin B was noncompetitive with *K*_i_ values of 23.1, 33.8, and 38.3 µM, respectively. AChE inhibition by (2*R*)-pteroside B, (2*R*,3*R*)-pteroside C, (2*R*)-pterosin B, (2*S*,3*R*)-pterosin C, and (3*S*)-pteroside D was mixed-type with *K*_i_ values of 4.89, 8.13, 12.1, 16.3, and 23.1 µM, respectively, while (2*R*,3*R*)-pterosin C was a noncompetitive type inhibitor with a *K*_i_ value of 29.6 µM. BChE inhibition by (2*R*,3*R*)-pterosin C, (2*R*,3*R*)-pteroside C, (3*S*)-pteroside D, and (2*R*)-pteroside B was mixed-type with *K*_i_ values of 4.77, 9.62, 19.7, and 22.6 µM, respectively, while (2*S*,3*R*)-pterosin C and (2*R*)-pterosin B were noncompetitive inhibitors with *K*_i_ values of 29.9 and 53.5 µM, respectively. Thus, these results suggested that specific pterosin derivatives might be effective BACE1, AChE, and BChE inhibitors.

**Table 2:** Enzyme kinetics of pterosin derivatives based on Dixon plot and Lineweaver−Burk plot

Compounds	*K*_i_ and inhibition type					
	BACE1		AChE		BChE	
	*K*_i_ (µM)^a^	Inhibition type^b^	*K*_i_ (µM)^a^	Inhibition type^b^	*K*_i_ (µM)^a^	Inhibition type^b^
(2*R*)-Pterosin B	38.3	Noncompetitive	12.1	Mixed-type	53.5	Noncompetitive
(2*S*,3*R*)-Pterosin C	33.8	Noncompetitive	16.3	Mixed-type	29.9	Noncompetitive
(2*R*,3*R*)-Pterosin C	27.6	Mixed-type	29.6	Noncompetitive	4.77	Mixed-type
(2*R*)-Pteroside B	23.1	Noncompetitive	4.89	Mixed-type	22.6	Mixed-type
(2*R*,3*R*)-Pteroside C	12.6	Mixed-type	8.13	Mixed-type	9.62	Mixed-type
(3*S*)-Pteroside D	16.5	Mixed-type	23.1	Mixed-type	19.7	Mixed-type

*BACE1* β-site amyloid precursor protein cleaving enzyme 1, *AChE* acetylcholinesterase, *BChE* butyrylcholinesterase

^a^Determined by Dixon plot

^b^Determined by Dixon and Lineweaver−Burk plots (Supplementary Information 2)

### Molecular docking simulations for BACE1, AChE, and BChE

Several crystal structures are available for BACE1 and cholinesterases. We selected human PDBs based on wild-type structures, cocrystallized ligands and resolutions of the structures. X-ray crystal structures of BACE1 complexed with QUD (PDB code: 2WJO, resolution: 2.5 Å)^33^, AChE complexed with E2020 (PDB code: 4EY7, resolution: 2.35 Å)^34^, and BChE complexed with 3F9 (PDB code: 4TPK, resolution: 2.70 Å)^35^ were selected for docking. Initially, QUD, E2020, and 3F9 were extracted from crystal structures and redocked into the active sites of BACE1, AChE, and BChE, respectively. Subsequently, (2*R*,3*R*)-pteroside C, (3*S*)-pteroside D, (2*R*,3*R*)-pterosin C, (2*R*)-pteroside B, (2*S*,3*R*)-pterosin C and (2*R*)-pterosin B with the known mechanism of inhibition against BACE1, AChE and BChE were docked to determine their SAR. The docking results are summarized in Table 3. The SAR of the selected mixed-type and noncompetitive BACE1, AChE, and BChE inhibitors enabled the evaluation of novel binding site-directed inhibition of the enzymes by pterosins.

**Table 3:** Docking affinity scores and possible H-bond formation to the corresponding active sites of BACE1, AChE, and BChE by pterosin derivatives along with reported inhibitors

Compounds	Target enzymes	B.E. (kcal/mol)^a^	H-bonds interacting residues	Hydrophobic interacting residues	Other interactions
QUD^b^	BACE1	–7.59	Asp32, Asp228, Gly230	Leu30, Tyr71, Phe108, Val332	
E2020^b^	AChE	–10.28	Phe295	Trp86, Trp286, Tyr337, Phe338, Tyr341	Trp286, Tyr341 (π-sigma)
3F9^b^	BChE	–8.49		Ile69, Gly116, Trp231, Leu286, Ala328, Phe329, Tyr332	Asp70 (π-anion)
(2*R*,3*R*)-Pteroside C	BACE1	–7.27	Ser36, Asn37, Asp228, Thr231	Ala39, Val69, Trp76, Ile118, Arg128	Val69 (π-sigma)
	AChE	–7.49	Trp86, Asn87, Tyr124	Tyr72, Tyr124, Trp286, Tyr337, Phe338	
	BChE	–7.23	Gly78, Ser287, Tyr440	Trp82, Phe329, Tyr332, Trp430, His438	
(3*S*)-Pteroside D	BACE1	–6.93	Ser36, Asn37, Ile126, Asp228	Val69, Tyr71, Trp76, Arg128	
	AChE	–4.91	Tyr72, Asp74	Tyr72, Tyr124, Trp286, Phe297, Phe338, Tyr341	Trp286 (π-sigma)
	BChE	–6.59	Trp82, Ser287	Ala328, Phe329, Tyr332, Trp430, Met437, His438	
(2*R*,3*R*)-Pterosin C	BACE1	–4.84	Ser36, Asn37	Ala39, Val69, Trp76, Ile118, Arg128	Val69 (π-sigma)
	AChE	–5.01	Tyr72, Ser293	Tyr72, Trp286, Phe297, Tyr341	
	BChE	–6.52	Gly78, Gly117, Tyr440	Trp82, Phe329, Tyr332, Trp430, His438	
(2*R*)-Pteroside B	BACE1	–6.16	Asn37, Trp76, Ile126	Val69, Tyr71, Phe108	
	AChE	–7.90	Trp86, Asn87, Tyr124	Tyr72, Tyr124, Trp286, Tyr337, Phe338, Tyr341	
	BChE	–4.38	Ser287	Trp82, Phe329, Tyr332, Trp430, His438	
(2*S*,3*R*)-Pterosin C	BACE1	–5.07	Lys107	Val69, Tyr76, Lys107, Phe108	
	AChE	–6.03	Tyr124, Phe295	Tyr72, Tyr124, Trp286, Tyr337, Phe338, Tyr341	
	BChE	–5.40	Gly283, Asn397	Leu286, Val288, Phe357	
(2*R*)-Pterosin B	BACE1	–4.64		Val69, Tyr76, Phe108	
	AChE	–5.76	Tyr124	Tyr124, Trp286, Tyr337, Phe338, Tyr341	
	BChE	–5.06	Gly283	Leu286, Val288, Phe357	

*B.E.* binding energy, *BACE1* β-site amyloid precursor protein cleaving enzyme 1, *AChE* acetylcholinesterase, *BChE* butyrylcholinesterase

^a^Estimated the binding free energy of the ligand receptor complex

^b^Positive control ligands

Our docking mode of E2020 was consistent with the experimentally determined binding mode previously reported with recombinant human AChE (rhAChE) (Supplementary Information 3)^34^. The root-mean-square deviation (RMSD) between the crystal and docked conformations of E2020 was 0.54 Å, which suggested the reliability of our docking setup in reproducing the experimental binding mode. In addition, the docked mode of E2020 led to a similar interaction as that of rhAChE-E2020. In our study, water molecules were removed from the crystal structure during docking; therefore, water-mediated interactions were not analyzed in the present study. Similarly, the docked modes of QUD and 3F9 were consistent with the available experimental data for BACE1 ^33^ and BChE^35^, respectively (Supplementary Information 3). The RMSDs between the crystal and docked conformations of QUD and 3F9 were 0.46 and 0.60 Å, respectively. Further, the binding sites of pterosin inhibitors were in agreement with a previous docking study that involved BACE1, AChE, and BChE^38^. However, the study used *Tetronarce californica* AChE (PDB code: 1ACJ), which contains slightly different residue numbers than human AChE due to variations in their sequences.

#### BACE1 docking

**Fig. 3:**
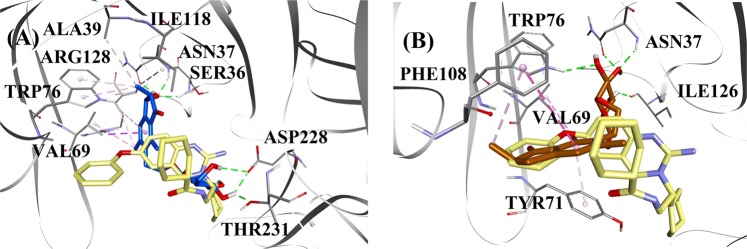
Molecular docking models for the mixed-type and the noncompetitive BACE1 inhibitors. Molecular docking models for **a** the mixed-type BACE1 inhibitor (2*R*,3*R*)-pteroside C (blue color) and **b** the noncompetitive BACE1 inhibitor (2*R*)-pteroside B (brown color). Docked poses are superimposed on the X-ray crystal structure of QUD (yellow color) (PDB code: 2WJO). BACE1, active site residues and compounds are shown by ribbon, line and stick models, respectively. Colors of the dotted lines explain the types of various interactions: hydrogen bonding interactions (green), hydrophobic interactions (pink) and π-sigma interactions (purple). BACE1 β-site amyloid precursor protein cleaving enzyme 1

**Fig. 4:**
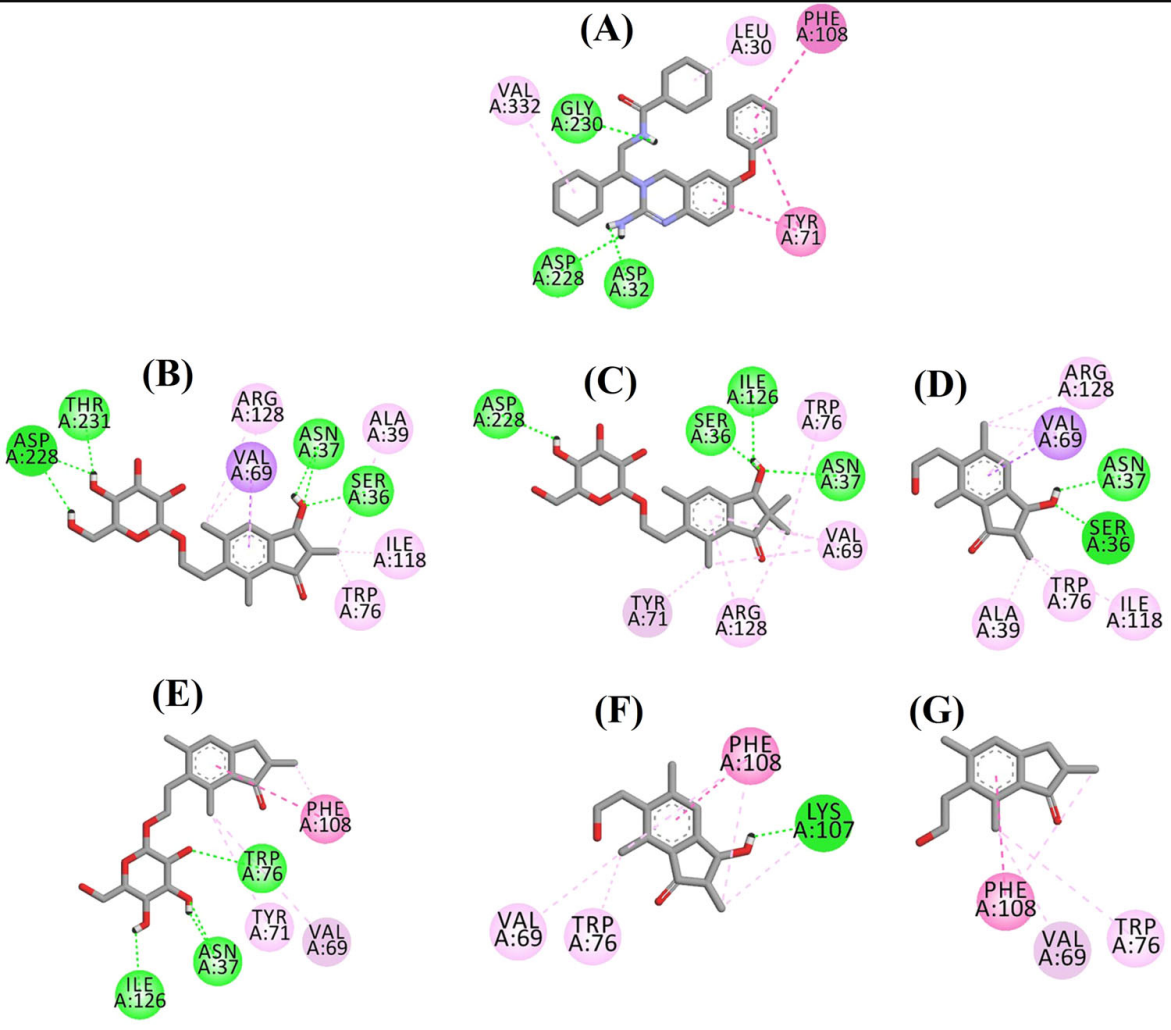
Ligand interaction diagram of BACE1 inhibitors in the active site. Ligand interaction diagram of **a** QUD, **b** (2*R*,3*R*)-pteroside C, **c** (3*S*)-pteroside D, **d** (2*R*,3*R*)-pterosin C, **e** (2*R*)-pteroside B, **f** (2*S*,3*R*)-pterosin C, and **g** (2*R*)-pterosin B in the active site of BACE1. Colors of the dotted lines explain the types of various interactions: hydrogen bonding interactions (green), hydrophobic interactions (pink) and π-sigma interactions (purple). BACE1 β-site amyloid precursor protein cleaving enzyme 1

Based on the inhibition type and activity, (2*R*,3*R*)-pteroside C and (2*R*)-pteroside B were selected to demonstrate the docked modes of mixed-type and noncompetitive BACE1 inhibitors, respectively. Figure 3a, b displays the docking models of (2*R*,3*R*)-pteroside C and (2*R*)-pteroside B, respectively. The interactions of the docked compounds inside the active site of BACE1 are shown in Fig. 4.

The docked pose of QUD exhibited a binding energy (B.E.) of −7.59 kcal/mol. As shown in Fig. 4a, the NH_2_ group on the quinazoline ring of the ligand showed two hydrogen bonds with the CO groups of Asp32 and Asp228 at distances of 1.86 and 2.17 Å, respectively. A third hydrogen bond was observed between the other NH group of the ligand and the CO group of Gly230 at a distance of 2.16 Å. Leu30, Tyr71, Phe108, and Val332 mediated the hydrophobic interactions. Figure 4b−d displays the docked poses of (2*R*,3*R*)-pteroside C, (3*S*)-pteroside D and (2*R*,3*R*)-pterosin C (mixed-type BACE1 inhibitors), respectively. They were positioned in the binding pocket lined by Ser36, Asn37, Ala39, Val69, Tyr71, Trp76, Ile118, Ile126, Arg128, Asp228, and Thr231. As per their activity levels, (2*R*,3*R*)-pteroside C (IC_50_ = 9.74 µM), (3*S*)-pteroside D (IC_50_ = 10.7 µM) and (2*R*,3*R*)-pterosin C (IC_50_ = 26.2 µM) exhibited a B.E. of −7.27, −6.93, and −4.84 kcal/mol, respectively. (2*R*,3*R*)-Pteroside C exhibited a higher potency than (2*R*,3*R*)-pterosin C due to the existence of an additional 2-hydroxymethyl-tetrahydro-pyran-3,4,5-triol group, which formed three hydrogen bonds (Fig. 4b). The OH group of hydroxymethyl and the 3-OH group of the tetrahydro-pyran-triol ring showed two hydrogen bonds with the CO group of Asp228 at distances of 2.63 and 2.12 Å, respectively. Additionally, the 3-OH group demonstrated a hydrogen bond with Thr231 at a distance of 1.89 Å. In the case of (3*S*)-pteroside D (Fig. 4c), the presence of the 2,2-dimethyl group at the indanone ring slightly altered the binding interactions compared with (2*R*,3*R*)-pteroside C. The 3-OH group of the tetrahydro-pyran-triol ring showed only a single hydrogen bond with Asp228 at a distance of 1.93 Å. However, the 3-OH group of the indanone ring exhibited an additional hydrogen bond with Ile126 at a distance of 2.34 Å. These interactions slightly lowered the activity of (3*S*)-pteroside D compared with (2*R*,3*R*)-pteroside C. The docking interactions of (2*R*,3*R*)-pterosin C displayed in Fig. 4d show the 3-OH group of the indanone ring bound to Ser36 and Asn37 via two hydrogen bonds at distances of 1.83 and 2.07 Å, respectively. Ala39, Val69, Trp76, Ile118, and Arg128 were involved in hydrophobic interactions, while Val69 displayed a π-sigma interaction.

The docked poses of (2*R*)-pteroside B, (2*S*,3*R*)-pterosin C, and (2*R*)-pterosin B (noncompetitive BACE1 inhibitors) are shown in Fig. 4e−g, respectively. They were docked into the cavity enclosed by Asn37, Val69, Tyr71, Trp76, Lys107, Phe108, and Ile126. In accordance with their activity values, (2*R*)-pteroside B (IC_50_ = 18.0 µM), (2*S*,3*R*)-pterosin C (IC_50_ = 23.1 µM), and (2*R*)-pterosin B (IC_50_ = 29.6 µM) exhibited a B.E. of −6.16, −5.07, and −4.64 kcal/mol, respectively. As displayed in Fig. 4e, (2*R*)-pteroside B demonstrated higher activity than (2*S*,3*R*)-pterosin C and (2*R*)-pterosin B due to the presence of an additional 2-hydroxymethyl-tetrahydro-pyran-3,4,5-triol group, which showed four hydrogen bond interactions. Two hydrogen bonds were observed between the 4-OH group of the tetrahydro-pyran-triol ring and the NH and CO groups of Asn37 at distances of 2.48 and 2.19 Å, respectively. Further, the 3- and 5-OH groups showed two additional hydrogen bonds with Ile126 and Trp76 at distances of 2.19 and 2.46 Å, respectively. (2*S*,3*R*)-Pterosin C showed slightly better activity than (2*R*)-pterosin B due to the presence of an additional OH group at position-3 of the indanone ring, which formed a hydrogen bond with Lys107 at a distance of 2.12 Å (Fig. 4f). The other interactions were similar to those of (2*R*)-pterosin B. As displayed in Fig. 4g, (2*R*)-pterosin B showed hydrophobic interactions with Val69, Tyr76, and Phe108.

#### AChE docking

**Fig. 5:**
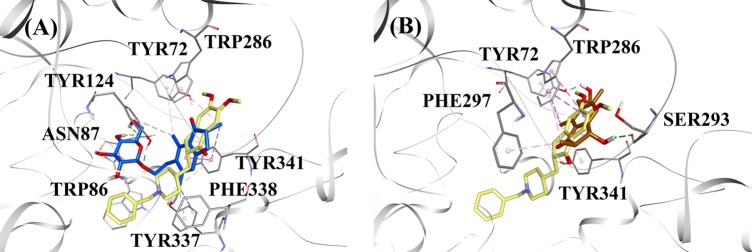
Molecular docking models for the mixed-type and the noncompetitive AChE inhibitors. Molecular docking models for **a** the mixed-type AChE inhibitor (2*R*)-pteroside B (blue color) and **b** the noncompetitive AChE inhibitor (2*R*,3*R*)-pterosin C (brown color). Docked poses are superimposed on the X-ray crystal structure of E2020 (yellow color) (PDB code: 4EY7). AChE, active site residues and compounds are shown by ribbon, line and stick models, respectively. Colors of the dotted lines explain the types of various interactions: hydrogen bonding interactions (green) and hydrophobic interactions (pink). AChE acetylcholinesterase

**Fig. 6:**
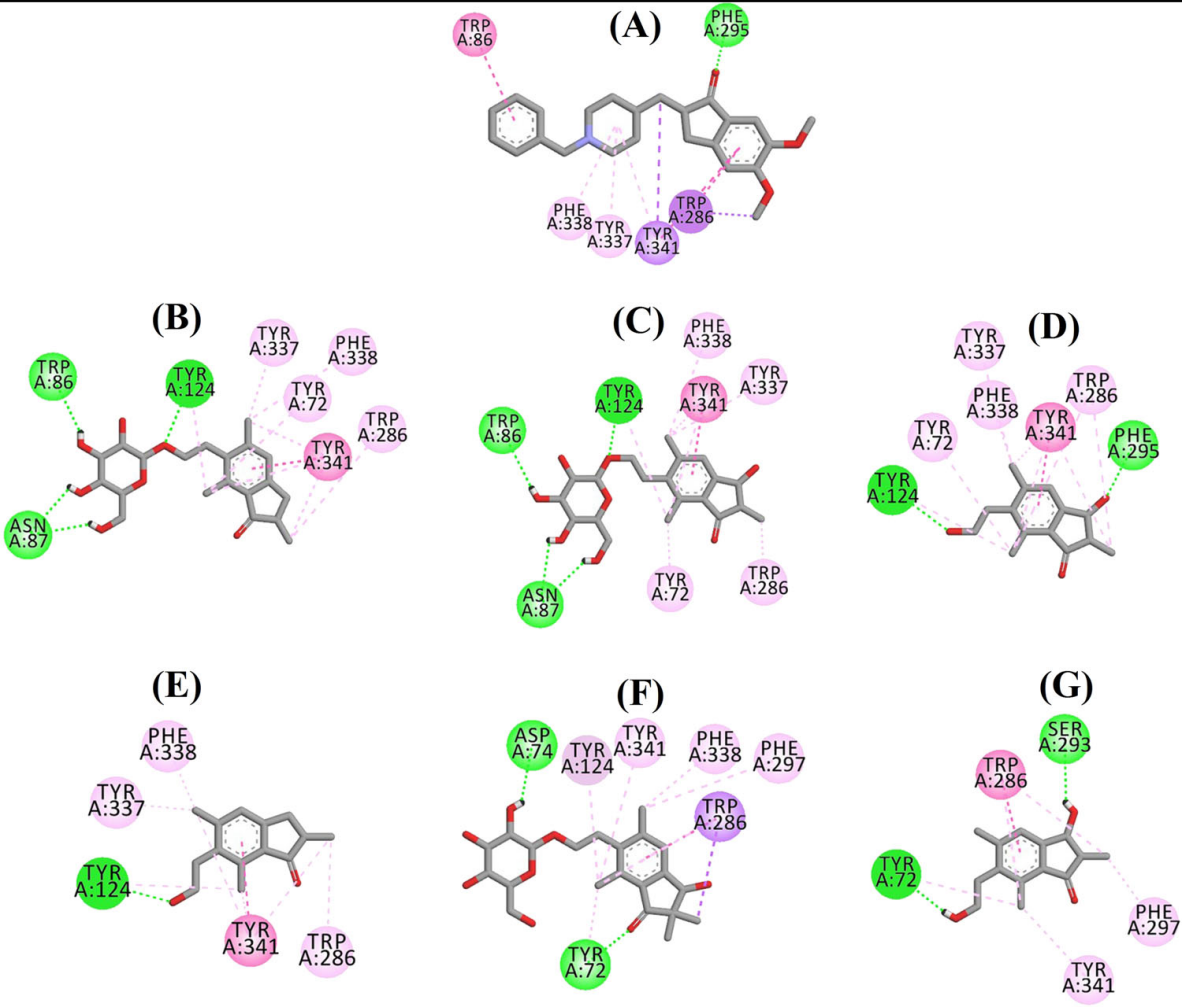
Ligand interaction diagram of AChE inhibitors in the active site. Ligand interaction diagram of **a** E2020, **b** (2*R*)-pteroside B, **c** (2*R*,3*R*)-pteroside C, **d** (2*S*,3*R*)-pterosin C, **e** (2*R*)-pterosin B, **f** (3*S*)-pteroside D and **g** (2*R*,3*R*)-pterosin C in the active site of AChE. Colors of the dotted lines explain the types of various interactions: hydrogen bonding interactions (green), hydrophobic interactions (pink) and π-sigma interactions (purple). AChE acetylcholinesterase

(2*R*)-Pteroside B and (2*R*,3*R*)-pterosin C were selected as representatives to demonstrate the docked modes of mixed-type and noncompetitive AChE inhibitors, respectively, due to their activities and type of AChE inhibition. Figure 5a, b illustrates the docking models of (2*R*)-pteroside B and (2*R*,3*R*)-pterosin C, respectively. The interactions of the docked compounds inside the active site of AChE are displayed in Fig. 6.

The docked pose of E2020 demonstrated a B.E. of −10.28 kcal/mol. As illustrated in Fig. 6a, the CO group of the indanone ring formed a hydrogen bond with the NH group of Phe295 at a distance of 1.70 Å. Trp286 and Tyr341 were involved in π-sigma interactions, whereas Trp86, Trp286, Tyr337, Phe338, and Tyr341 mediated hydrophobic interactions. Figure 6b−f demonstrates the docked poses of (2*R*)-pteroside B, (2*R*,3*R*)-pteroside C, (2*S*,3*R*)-pterosin C, (2*R*)-pterosin B and (3*S*)-pteroside D (mixed-type AChE inhibitors), respectively. They were accommodated in the active site surrounded by Tyr72, Asp74, Trp86, Asn87, Tyr124, Trp286, Phe295, Phe297, Tyr337, Phe338, and Tyr341. Consistent with their activity values, (2*R*)-pteroside B (IC_50_ = 2.55 µM), (2*R*,3*R*)-pteroside C (IC_50_ = 3.77 µM), (2*S*,3*R*)-pterosin C (IC_50_ = 12.8 µM), (2*R*)-pterosin B (IC_50_ = 16.2 µM) and (3*S*)-pteroside D (IC_50_ = 27.4 µM) exhibited a B.E. of −7.90, −7.49, −6.03, −5.76, and −4.91 kcal/mol, respectively. (2*R*)-Pteroside B demonstrated a higher potency than (2*S*,3*R*)-pterosin C and (2*R*)-pterosin B due to the presence of an additional 2-hydroxymethyl-tetrahydro-pyran-3,4,5-triol group, which established three hydrogen bond interactions (Fig. 6b). The OH group of hydroxymethyl and the 3-OH group of the tetrahydro-pyran-triol ring displayed hydrogen bonds with the CO group of Asn87 at distances of 2.23 and 2.28 Å, respectively. Further, the 4-OH group formed a hydrogen bond with the CO group of Trp86 at a distance of 2.13 Å. In the case of (2*R*,3*R*)-pteroside C (Fig. 6c), the methyl group at position-2 of the indanone ring did not show a hydrophobic interaction with Tyr341 and thus exhibited comparatively lower activity than (2*R*)-pteroside B. However, Tyr341 maintained the hydrophobic interaction with the other part of the indanone ring as shown in that of (2*R*)-pteroside B. The higher activity of (2*S*,3*R*)-pterosin C than (2*R*)-pterosin B was attributed to the existence of an additional OH group at position-3 of the indanone ring, which formed a hydrogen bond with Phe295 at a distance of 1.82 Å (Fig. 6d). The remaining interactions were comparable to (2*R*)-pterosin B interactions. As shown in Fig. 6e, the OH group of the hydroxyethyl group at position-6 of the indanone ring formed a hydrogen bond with Tyr124 at a distance of 2.48 Å. Tyr124, Trp286, Tyr337, Phe338, and Tyr341 contributed to the hydrophobic interactions. Compared with (2*R*)-pteroside B and (2*R*,3*R*)-pteroside C, (3*S*)-pteroside D exhibited dissimilar binding interactions due to the presence of the 2,2-dimethyl group at the indanone ring (Fig. 6f). The 2,2-dimethyl group significantly contributed to the distinct docked pose of (3*S*)-pteroside D. The 5-OH group of the tetrahydro-pyran-triol ring formed a hydrogen bond with Asp74 at a distance of 1.96 Å. The CO group of the indanone ring showed a hydrogen bond with Tyr72 at a distance of 2.97 Å. These interactions accounted for the low activity of (3*S*)-pteroside D.

The docked pose of (2*R*,3*R*)-pterosin C (noncompetitive AChE inhibitor) is displayed in Fig. 6g. (2*R*,3R)-Pterosin C (IC_50_ = 23.2 µM) demonstrated a B.E. of −5.01 kcal/mol. The binding pocket of (2*R*,3*R*)-pterosin C comprised Tyr72, Trp286, Ser293, Phe297, and Tyr341, with two hydrogen bond interactions. One of the hydrogen bonds was formed between the 3-OH group of the indanone ring and the CO group of Ser293 at a distance of 2.03 Å. The second hydrogen bond was observed between the OH group of the hydroxyethyl group present at position-6 of the indanone ring and Tyr72 at a distance of 1.94 Å. Residues such as Tyr72, Trp286, Phe297, and Tyr341 participated in hydrophobic interactions.

#### BChE docking

**Fig. 7:**
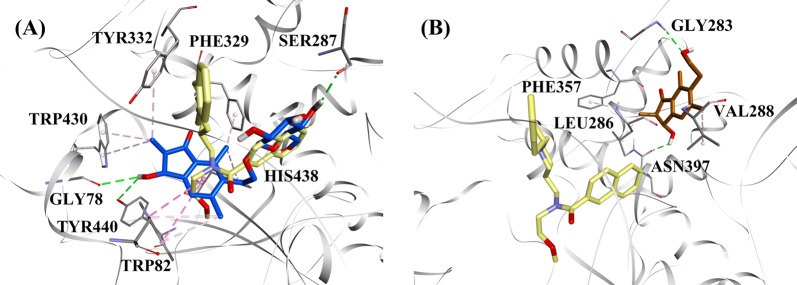
Molecular docking models for the mixed-type and the noncompetitive BChE inhibitors. Molecular docking models for **a** the mixed-type BChE inhibitor (2*R*,3*R*)-pteroside C (blue color) and **b** the noncompetitive BChE inhibitor (2*S*,3*R*)-pterosin C (brown color). Docked poses are superimposed on the X-ray crystal structure of 3F9 (yellow color) (PDB code: 4TPK). BChE, active site residues and compounds are shown by ribbon, line and stick models, respectively. Colors of the dotted lines explain the types of various interactions: hydrogen bonding interactions (green) and hydrophobic interactions (pink). BChE butyrylcholinesterase

**Fig. 8:**
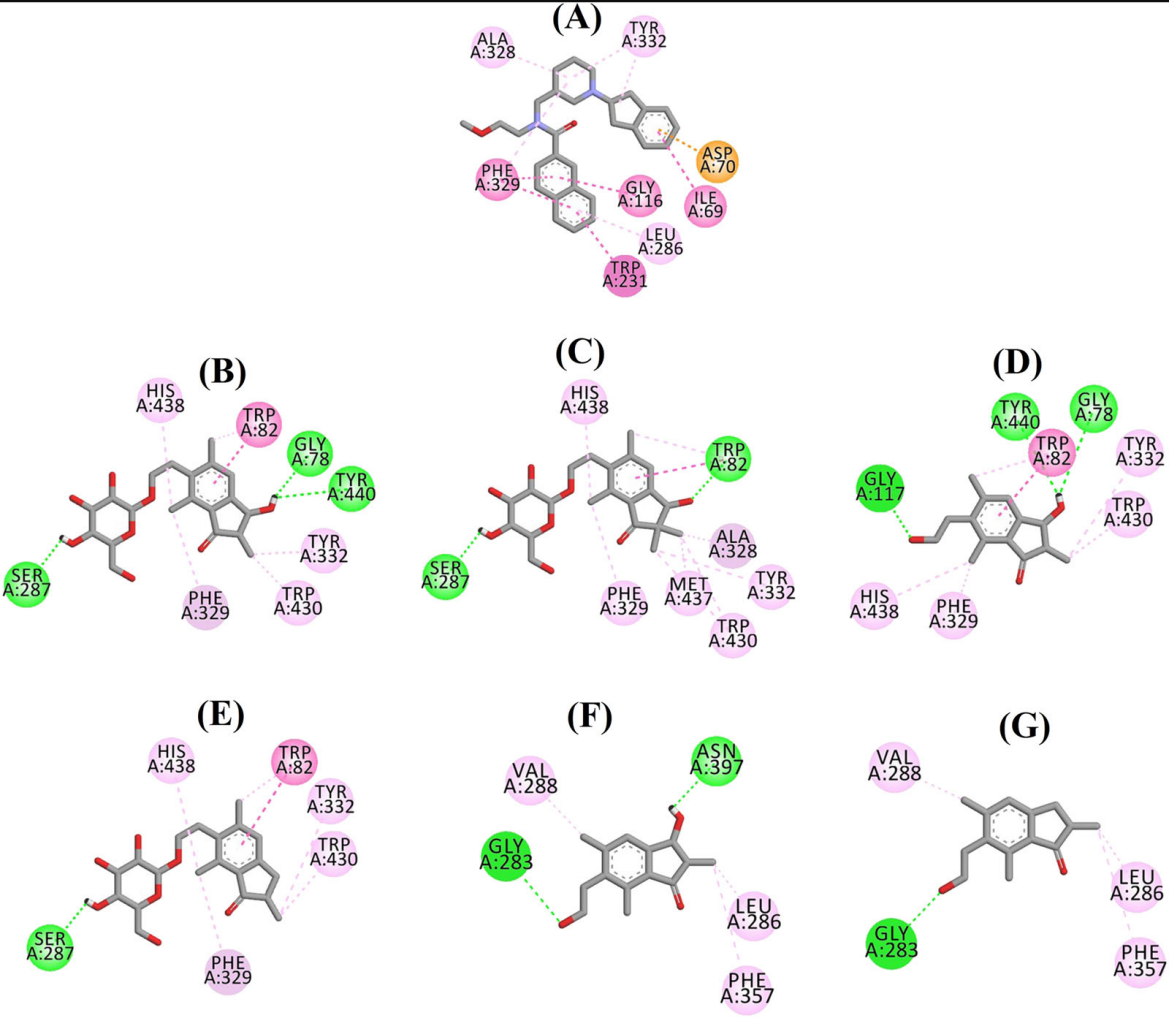
Ligand interaction diagram of BChE inhibitors in the active site. Ligand interaction diagram of **a** 3F9, **b** (2*R*,3*R*)-pteroside C, **c** (3*S*)-pteroside D, **d** (2*R*,3*R*)-pterosin C, **e** (2*R*)-pteroside B, **f** (2*S*,3*R*)-pterosin C and **g** (2*R*)-pterosin B in the active site of BChE. Colors of the dotted lines explain the types of various interactions: hydrogen bonding interactions (green), hydrophobic interactions (pink) and π-anion interactions (golden). BChE butyrylcholinesterase

Considering the activity levels and type of BChE inhibition, (2*R*,3*R*)-pteroside C and (2*S*,3*R*)-pterosin C were selected to demonstrate the docked modes of mixed-type and noncompetitive BChE inhibitors, respectively. Figure 7a, b illustrates the docking models of (2*R*,3*R*)-pteroside C and (2*S*,3*R*)-pterosin C, respectively. The interactions of the docked compounds inside the BChE active site are presented in Fig. 8.

The docked pose of 3F9 showed a B.E. of −8.49 kcal/mol. As displayed in Fig. 8a, hydrophobic interactions were mainly responsible for the ligand binding. Ile69, Gly116, Trp231, Leu286, Ala328, Phe329, and Tyr332 accounted for the hydrophobic interactions, while Asp70 demonstrated a π-anion interaction. Figure 8b−e illustrates the docked poses of (2*R*,3*R*)-pteroside C, (3*S*)-pteroside D, (2*R*,3*R*)-pterosin C and (2*R*)-pteroside B (mixed-type BChE inhibitors), respectively. Their binding pocket was composed of Gly78, Trp82, Gly117, Ser287, Ala328, Phe329, Tyr332, Trp430, Met437, His438, and Tyr440. In accordance with their activity levels, (2*R*,3*R*)-pteroside C (IC_50_ = 5.29 µM), (3*S*)-pteroside D (IC_50_ = 19.3 µM), (2*R*,3*R*)-pterosin C (IC_50_ = 20.3 µM) and (2*R*)-pteroside B (IC_50_ = 62.0 µM) demonstrated a B.E. of −7.23, −6.59, −6.52, and −4.38 kcal/mol, respectively. As shown in Fig. 8b, the 3-OH group of the indanone ring of (2*R*,3*R*)-pteroside C showed two hydrogen bonds with Gly78 and Tyr440 at distances of 2.87 and 2.89 Å, respectively. In the case of (3*S*)-pteroside D, the 2,2-dimethyl group at the indanone ring affected the binding interactions of the 3-OH group (Fig. 8c). The 3-OH group formed only one hydrogen bond with Trp82 at a distance of 2.94 Å, which resulted in a comparatively lower activity of (3*S*)-pteroside D than (2*R*,3*R*)-pteroside C. As shown in Fig. 8d, (2*R*,3*R*)-pterosin C failed to produce a hydrogen bond with Ser287 due to the absence of a 2-hydroxymethyl-tetrahydro-pyran-3,4,5-triol group. Consequently, it yielded a lower activity than (2*R*,3*R*)-pteroside C. The lack of the OH group at position-3 of the indanone ring was found to be responsible for the very low activity of (2*R*)-pteroside B (Fig. 8e), which failed to form hydrogen bonds with Gly78 and Tyr440 similar to (2*R*,3*R*)-pteroside C.

The docked poses of (2*S*,3*R*)-pterosin C and (2*R*)-pterosin B (noncompetitive BChE inhibitors) are shown in Fig. 8f, g, respectively. These docked poses were contained in the cavity enclosed by Gly283, Leu286, Val288, Phe357, and Asn397. As per their activity levels, (2*S*,3*R*)-pterosin C (IC_50_ = 44.3 µM) and (2*R*)-pterosin B (IC_50_ = 48.1 µM) exhibited a B.E. of −5.40 and −5.06 kcal/mol, respectively. (2*S*,3 *R*)-Pterosin C was more potent than (2*R*)-pterosin B because of the presence of an additional OH group at position-3 of the indanone ring, which formed a hydrogen bond with the CO group of Asn397 at a distance of 2.05 Å (Fig. 8f). Other interactions were found to be similar to (2*R*)-pterosin B. As shown in Fig. 8g, the OH group in the hydroxyethyl group at position-6 of the indanone ring formed a hydrogen bond with Gly283 at a distance of 2.40 Å. The residues Leu286, Val288, and Phe357 participated in hydrophobic interactions.

Mixed-type inhibitors bind to both the free enzyme and the enzyme-substrate complex, which indicates that these compounds may bind to the catalytic site of each corresponding enzyme. Noncompetitive inhibitors bind to the allosteric site of the free enzyme or enzyme-substrate complex. A recent study suggested that competitive, mixed-type and noncompetitive inhibitors occupy different sites in the binding pockets of BACE1, AChE, and BChE^38^. During docking for the evaluation of the inhibitory mechanism of pterosin derivatives, the binding sites of the compounds were defined according to their type of inhibition. The docking results indicated that the binding sites of mixed-type and noncompetitive inhibitors for BACE1, AChE, and BChE partially overlap each other at each corresponding active site and were consistent with a previous report^38^.

### BBB permeability

PAMPA-BBB, an in vitro artificial membrane permeability assay for the BBB, is one of the most reliable physicochemical screening tools in the early stage discovery of CNS-targeted drugs^40^. The PAMPA-BBB system models the transcellular passive diffusion of chemicals across the BBB and measures strictly passive transport mechanisms via an artificial lipid membrane on effective permeability (*P*_e_, cm/s). On the basis of the pattern established for BBB permeation prediction, compounds were classified into (i) “CNS+” (high BBB permeation predicted); *P*_e_ (10^−6^ cm/s) > 4.00, (ii) “CNS−” (low BBB permeation predicted); *P*_e_ (10^−6^ cm/s) < 2.00, and (iii) “CNS+/−” (BBB permeation uncertain); *P*_e_ (10^−6^ cm/s) from 4.00 to 2.00. Accordingly, (2*R*)-pterosin B, (2*S*)-pterosin P, and (2*S*)-pterosin A exhibited high BBB permeation with *P*_e_ values of 60.3 × 10^−6^ cm/s, 7.92 × 10^−6^ cm/s, and 6.26 × 10^−6^ cm/s, respectively (Table 4, Supplementary Information 4). The *P*_e_ value of (2*R*)-pterosin B was 1.7-fold higher than that of the CNS drug verapamil (*P*_e_ = 34.6 × 10^−6^ cm/s), which was used for the positive control. (2*S*,3*R*)-Pterosin C and (2*R*,3*R*)-pterosin C showed uncertain BBB permeation with *P*_e_ values of 2.34 and 1.98, respectively. (2*R*,3*R*)-Pteroside C, (3*S*)-pteroside D, and (2*R*)-pteroside B, which showed the most potent BACE1- and cholinesterase-inhibitory activities among the pterosin derivatives tested, exhibited a very low BBB permeability. The existence of the 2-hydroxymethyl-tetrahydro-pyran-3,4,5-triol group as in pteroside derivatives resulted in a remarkable decrease in the BBB permeability. Compared with (2*R*)-pterosin B, the additional presence of the OH group at position-3 of the indanone ring as in pterosin C, the hydroxymethyl group at position-2 of the indanone ring as in (2*S*)-pterosin A or the hydroxymethyl group at position-5 of the indanone ring as in (2*S*)-pterosin P also significantly reduced the BBB permeability. Considering an exceptionally high BBB permeability and the significant inhibition of BACE1, AChE, and BChE, (2*R*)-pterosin B may have the potential to exhibit a strong anti-AD activity.

**Table 4:** PAMPA-BBB permeability of pterosin derivatives

Compounds	PAMPA-BBB permeability *P*_e_ (10^−6^ cm/s)
(2*S*)-Pterosin A	6.26 ± 0.24
(2*R*)-Pterosin B	60.3 ± 9.8
(2*S*,3 *R*)-Pterosin C	2.34 ± 0.81
(2*R*,3 *R*)-Pterosin C	1.98 ± 0.17
(2*S*)-Pterosin P	7.92 ± 0.36
(2*S*)-Pteroside A_2_	0.54 ± 0.51
(2*R*)-Pteroside B	0.48 ± 0.29
(2*S*,3*R*)-Pteroside C	0.29 ± 0.34
(2*R*,3*R*)-Pteroside C	0.00 ± 0.00
(3*S*)-Pteroside D	0.00 ± 0.00
Pteroside Z	0.61 ± 0.17
Verapamil^a^	34.6 ± 3.9

PAMPA-BBB permeability *P*_e_ (10^−6^ cm/s) is expressed as the mean ± SD of quadruple experiments (Supplementary Information 4)

“CNS+” (high BBB permeation predicted); *P*_e_ > 4.0, “CNS+/−” (BBB permeation uncertain); *P*_e_ from 4.0 to 2.0, “CNS−” (low BBB permeation predicted); *P*_e_ < 2.0^40^

*PAMPA* parallel artificial membrane permeation assay

^a^Verapamil was used as positive control

### Effects of (2*R*)-pterosin B and (2*R*,3*R*)-pteroside C on the secretion of Aβ peptides by neuronal cells

**Fig. 9:**
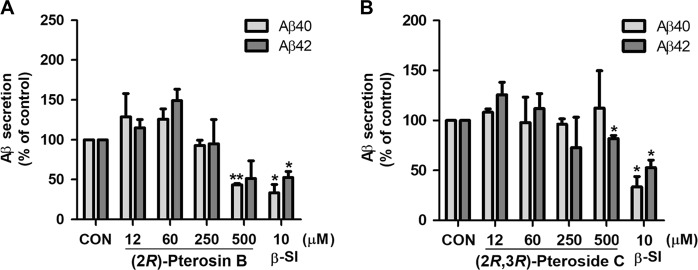
The effects of (2*R*)-pterosin B and (2*R*,3*R*)-pteroside C on the cellular secretion of Aβ peptides. **a** Effect of (2*R*)-pterosin B on the secretion of Aβ peptides. APPswe-secreting neuroblastoma cells were treated with 12, 60, 250, or 500 µM of (2*R*)-pterosin B for 24 h, and conditioned media were collected in the presence of protease inhibitor. β-SI (10 µM) was used as positive control. Negative control included cells cultured in the absence of test compounds. Quantitative analysis of secreted Aβ40 and Aβ42 in the conditioned media was performed using sandwich ELISA. The means ± SD from three independent experiments are shown. The secreted Aβ40 peptides significantly decreased in the presence of 500 µM (2*R*)-pterosin B. CON negative control, β-SI β-secretase inhibitor III, Aβ β-amyloid. *P < 0.05, **P < 0.01. **b** Effect of (2*R*,3*R*)-pteroside C on the secretion of Aβ peptides. APPswe-secreting neuroblastoma cells were treated with 12, 60, 125, 250, or 500 µM of (2*R*,3*R*)-pteroside C for 24 h, and conditioned media were collected in the presence of protease inhibitor. β-SI (10 µM) was used as positive control. Negative control included cells cultured in the absence of test compounds. Quantitative analysis of secreted Aβ40 and Aβ42 in the conditioned media was performed using sandwich ELISA. The means ± SD from three independent experiments are shown. The secreted Aβ42 peptide significantly decreased in the presence of 500 µM (2*R*,3*R*)-pteroside C. CON negative control, β-SI β-secretase inhibitor III, Aβ β-amyloid. *P < 0.05

To investigate the function of (2*R*)-pterosin B and (2*R*,3*R*)-pteroside C in decreasing the excretion of Aβ from neuronal cells, we used a murine neuroblastoma cell line that stably overexpresses human APPswe. The cell line is a cellular model of AD characterized by the excessive secretion of Aβ40 and Aβ42. Toxic amyloid oligomers are formed from the two isoforms of Aβ peptide with different lengths. Aβ40 is the most abundant Aβ isoform in the brain^41^, while Aβ42 significantly increases with certain forms of AD^42^. Sandwich ELISA of Aβ40 showed that (2*R*)-pterosin B significantly reduced the amount of Aβ40 peptide secreted from the neuroblastoma cells into media up to 50% at 500 μM (*P* < 0.01) (Fig. 9a). Similarly, the secretion of Aβ42 peptide by the neuroblastoma cells significantly decreased in the presence of 500 µM of (2*R*,3*R*)-pteroside C (*P* < 0.05) (Fig. 9b). In conclusion, (2*R*)-pterosin B and (2*R*,3*R*)-pteroside C significantly decreased the secretion of Aβ peptides from neuroblastoma cells at a concentration of 500 μM.

### Cytotoxicity of pterosin derivatives based on MTT assay^43^

Overall, pterosin derivatives displayed negligible cytotoxicity against various normal and cancer cell lines, such as SH-SY5Y human neuroblastoma, C6 rat glial cells, NIH3T3 mouse embryo fibroblasts and B16F10 mouse melanoma with LD_50_ values above 0.5 mM (Supplementary Information 5). In particular, (2*R*)-pterosin B, (3*R*)-pterosin D, (2*S*)-pterosin P, (2*S*)-pteroside A, (2*R*)-pteroside B, and (2*R*,3*R*)-pteroside C showed no cytotoxicity against the cell lines tested with LD_50_ values above 5 mM. Intriguingly, several pterosins showed relative antiproliferative effects against SH-SY5Y neuronal cells compared with C6 glial cells of mesenchymal origin. The present results were consistent with a previous report that suggested pterosin derivatives are nontoxic to humans^26^.

## Discussion

The current study is the first investigation to evaluate pterosin derivatives as a series of novel scaffolds to provide MTDLs, which displayed significant inhibitory activities against BACE1, AChE, and BChE simultaneously in a dose-dependent manner. The molecular structures of the enzyme/inhibitor complexes were further predicted to simulate binding between the pterosin derivatives and BACE1, AChE, and BChE. These predictions facilitated the evaluation of binding site-directed inhibition of the enzymes. Furthermore, the docking results explained the SAR of selected mixed-type and noncompetitive BACE1, AChE, and BChE inhibitors. Both the in vitro evaluation and molecular docking data clearly indicated that specific pterosin compounds are potential lead compounds for the development of novel MTDLs for AD therapeutics via the Aβ and cholinesterase pathways.

In particular, (2*R*)-pterosin B exhibited the highest BBB permeation among commercially available drugs currently used for CNS diseases based on *P*_e_ representing effective BBB permeability in vitro^40^. Therapeutic candidates for CNS diseases, including AD, must be able to permeate the BBB. Only compounds with a molecular weight smaller than 400–700 Da and lipophilicity have been shown to cross the BBB^44^. Compared with (2*R*)-pterosin B, the additional 2-hydroxymethyl-tetrahydro-pyran-3,4,5-triol group as in pteroside derivatives, the presence of the 3-OH group as in pterosin C or the hydroxymethyl group at position-2 or 5 of the indanone ring as in (2*S*)-pterosin A or (2*S*)-pterosin P remarkably decreased the effective BBB permeability.

The present experiment used quercetin and berberine as positive controls for the BACE1 and cholinesterase assays, respectively. The flavonoid quercetin is a BACE1 inhibitor that exhibits novel pharmacophore features for AD^45^, as well as an inhibitor for AChE (IC_50_ = 19.8 µM)^46^ with a negligible influence on BChE activity^47^; moreover, it effectively ameliorated AD pathology and tauopathy and protected cognitive and emotional functions in aged (21–24 months old) triple transgenic mouse models of AD (3xTg-AD) treated with 25 mg/kg via i.p. injection every 48 h for 3 months^48^. Quercetin was reported to exhibit effective BBB permeability, with a log *P*_e_ of approximately −7 based on PAMPA-BBB^49^. Moreover, (2*R*)-pterosin B displayed BBB permeability with a log *P*_e_ of −4.22, which represented an approximately 600-fold higher *P*_e_ than that of quercetin. Considering that (2*R*)-pterosin B displayed a potency to inhibit BACE1 and AChE (IC_50_ = 29.6 and 16.2 µM, respectively) comparable to quercetin (IC_50_ = 18.8 µM and 19.8 µM, respectively) and a significant BChE-inhibitory activity (IC_50_ = 48.1 µM) that quercetin lacks along with an exceptionally high BBB permeability, (2*R*)-pterosin B was suggested to have a potential to ameliorate AD symptoms. Berberine exhibits a potent anticholinesterase activity^50^ and the ability to increase the cell viability in the hippocampus and peripheral neurons by enhancing remyelination of neuronal cells^51^, as well as increases the synthesis of interleukin-Iβ and inducible nitric oxide synthase in a rat model of AD^52^. Berberine demonstrated procognitive and antiamnestic properties in dementia animal models treated with 5 mg/kg via i.p. injection^53^ and efficient neuroprotection by reducing the permeability of leukocytes to the injury site in a mouse model of traumatic brain injury^54^. The log BB of berberine, a pharmacokinetic descriptor of the brain, was reported to be −0.35^53^, which corresponds to a log *P*_e_ of −5.8 according to the correlation between the experimental log BB value and the effective permeability, *P*_e_, determined by PAMPA-BBB, log BB = 0.612 × log *P*_e_ + 3.206, *R*^2^ = 0.723^49^. (2*R*)-Pterosin B exhibited a log *P*_e_ of −4.22, which represented an approximately 37-fold higher *P*_e_ than that of berberine along with the BACE1-inhibitory activity that berberine lacks^55^, although the AChE- and BChE-inhibitory activities of (2*R*)-pterosin B (IC_50_ against AChE = 16.2 µM, BChE = 48.1 µM, respectively) were lower than those of berberine (IC_50_ against AChE = 0.39 µM, BChE = 3.32 µM, respectively) approximately 40-fold and 15-fold, respectively.

The main disadvantage of MTDLs as hybrid molecules is their high molecular weight, which conforms to Lipinski’s rule of five^56^. Thus, the development of merged ligands with a small molar mass similar to a single compound is more difficult and tedious^16,22,23^. Despite the obstacles, several successful drug scaffolds that contain merged ligands addressing cholinesterases and Aβ oligomerization simultaneously have recently been reported. Bis-tacrines bearing a peptide moiety that specifically prevents surface sites of human AChE from Aβ binding were developed as MTDLs to combat AD^57^. Accordingly, the hybrid compounds bind the catalytic and peripheral sites of human AChE and act as potent inhibitors of both the catalytic and noncatalytic functions of AChE, interfering with the Aβ self-oligomerization process via its peripheral anionic site^58,59^. Further, the inhibition of both human BChE and fatty acid amide hydrolase (FAAH) as dual cholinesterase-FAAH inhibitors that target both cholinergic and endocannabinoid signaling resulted in improved neuronal transmission by ACh and a simultaneous reduction of neuroinflammation^60^. (2*R*)-Pterosin B exhibited significant inhibitory activities against BACE1, AChE, and BChE along with a remarkably high in vitro BBB permeability, which suggests the potential as a scaffold for MTDLs to suppress Aβ production and simultaneously enhance cholinesterase-mediated cognitive functions. Intriguingly, (2*R*)-pterosin B was demonstrated to activate CREB signaling to protect cartilage against osteoarthritic change^28^. Moreover, the metabolic pathways involving cyclic nucleotides, including cAMP and cGMP, play an important role in the pathogenesis of AD via CREB activation, which has been regarded as a molecular switch required for learning, memory and neuronal survival^7,61–63^. The role of (2*R*)-pterosin B in the activation of CREB signaling and its effects on cognitive functions and neuroprotection in the brain merit evaluation.

Importantly, the present cytotoxicity test based on the MTT assay supported prior observations that pterosin derivatives were nontoxic to humans and not carcinogenic^26^, although some are cytotoxic to cancer cells, such as HeLa cells^64^. Further, previous animal experiments demonstrated the biosafety of pterosin derivatives. A mouse model of osteoarthritis was injected with a large amount of pterosin B (15 μL of 900 μM pterosin B) into the intra-articular space of the knee joint three times per week for an extended period of 8–13 weeks, without adverse effects, while ameliorating osteoarthritis^28^. Further, pterosin A, administered orally in large amounts of 100 mg/kg/day for 4 weeks, displayed no significant adverse effects, while effectively improving glucose intolerance and insulin resistance in various mouse models of diabetes^29,30^. Additionally, the diabetes animal experiment demonstrated the good oral bioavailability of pterosin A. In our MTT-based cytotoxic test, (2*R*)-pterosin B exhibited biosafety comparable to (2*S*)-pterosin A (Supporting Information 5). Intriguingly, diabetes and insulin resistance have emerged as significant risk factors aggravating AD^65^. In this context, the antidiabetes potential of pterosin-based anti-AD agents is worth investigation.

Considerable genetic and molecular evidence supports that BACE1 is the rate-limiting enzyme in the production of Aβ and the crucial pathogenetic events that lead to AD^66^. Moreover, several AChE and BChE inhibitors, such as E2020, were approved for the treatment of mild to moderate AD, and the use of these drugs is beneficial in the treatment of AD symptoms^19–21^. Although the in vitro inhibitory capacities of (2*R*)-pterosin B against BACE1 (*K*_i_ = 38.3 µM), AChE (IC_50_ = 16.2 µM) and BChE (IC_50_ = 48.1 µM) are substantially lower than those of a BACE1 inhibitor, AZD3293 (*K*_i_ = 0.4 nM) in a clinical study^67^ or the AChE inhibitor E2020 (IC_50_ = 6.7 nM) approved for clinical use that share some structural features with (2*R*)-pterosin B^68^, we suggest the possibility that pterosin may be administered up to dosages to reach effective concentrations to inhibit BACE1, AChE, and BChE in the brain of AD patients because of its biosafety and significantly high BBB permeability.

In conclusion, the currently available therapies for AD are only symptomatic. The MTDL approach is a very promising strategy for the treatment of AD due to its multifactorial etiology^22,23^. The structures of several pterosins suggest a potential biological and therapeutic role as a scaffold to provide new MTDLs for AD. Further, our results suggested that the molecular features of enzyme binding and BBB permeability of pterosins facilitate the structural modifications for the design of compounds with improved enzyme-inhibitory activities and BBB permeability for consideration as novel therapeutics for AD.

## Supplementary information

Supplementary Information

## **Code availability**

Human BACE1, 2WJO; Human AChE, 4EY7; Human BChE, 4TPK; Tetronarce californica AChE, 1ACJ.
