# Supplementary material for: Inhibition of β-site amyloid precursor protein cleaving enzyme 1 and cholinesterases by pterosins via a specific structure−activity relationship with a strong BBB permeability
Source: Exp Mol Med. 2019 Feb 12;51(2):12. doi: 10.1038/s12276-019-0205-7 (PMC6372667; doi:10.1038/s12276-019-0205-7)
Supplement: Supplementary file 1 — Supplementary Information [file 12276_2019_205_MOESM1_ESM.docx]

**Supplementary Information**

**Inhibition of β-site amyloid precursor protein cleaving enzyme 1 and cholinesterases by pterosins via a specific structure-activity relationship with a strong BBB permeability**

Susoma Jannat^1^**^*^**, Anand Balupuri^2^**^*^**, Md Yousof Ali^1^, Seong Su Hong^3^, Chun Whan Choi^3^, Yun-

Hyeok Choi^3^, Jin-Mo Ku^3^, Woo Jung Kim^3^, Jae Yoon Leem^4^, Ju Eun Kim^4^, Abinash Chandra Shrestha^4^, Ha Neul Ham^4^, Kee-Ho Lee^5^, Dong Min Kim^6^, Nam Sook Kang^2^ and Gil Hong Park^1^

^1^Department of Biochemistry and Molecular Biology, College of Medicine, Korea Molecular Medicine and Nutrition Research Institute, Korea University, Seoul 02841, Korea

^2^Graduate School of New Drug Discovery and Development, Chungnam National University, Daejeon 34134, Korea

^3^Bio-Center, Gyeonggido Business & Science Accelerator, Suwon 16229, Korea

^4^College of Pharmacy, Woosuk University, Wanju, Jeonbuk 55338, Korea

^5^Division of Radiation Cancer Research, Korea Institute of Radiological and Biomedical Sciences, Seoul 01812, Korea

^6^Department of Creative Arts Psychotherapy, College of Cultural Convergence, Jeonju University, Jeonju 55069, Korea

**Table of Contents**

**List of Supplementary Information Page**

**Supplementary Information 1**

Characterization of pterosin compounds **1**-**15**………………..…………………………S3

**Supplementary Information 2**

Dixon and Lineweaver-Burk plots for BACE1 inhibition by pterosin derivatives……….S9

Dixon and Lineweaver-Burk plots for AChE inhibition by pterosin derivatives………...S11

Dixon and Lineweaver-Burk plots for BChE inhibition by pterosin derivatives………...S13

**Supplementary Information 3**

Crystal and docked conformations of E2020 (donepezil).…………………………..……S15

Crystal and docked conformations of QUD……………...………………..……………...S16

Crystal and docked conformations of 3F9……………………….…………….……..…..S17

**Supplementary Information 4**

BBB permeability of pterosin derivatives………………………………………………..S18

**Supplementary Information 5**

Cytotoxicity of pterosin derivatives ………………………………………………….…..S24

**Supplementary Information 1**

**Characterization of Pterosin Compounds 1-15**

**(2*R*)-pterosin B (1)**. White amorphous powder; ^1^H-NMR (700 MHz, CDCl_3_) δ 7.23 (1H, s, H-4), 3.89 (2H, t, *J* = 7.7 Hz, H-14), 3.36 (1H, dd, *J* = 16.8, 7.7 Hz, H_a_-3), 3.15 (2H, t, *J* = 7.7 Hz, H-13), 2.81 (3H, s, H-15), 2.75 (1H, m, H-2), 2.70 (1H, dd, *J* = 16.8, 4.2 Hz, H_b_-3), 2.58 (3H, s, H-12), 1.40 (3H, d, *J* = 7.0 Hz, H-11); ^13^C-NMR (175 MHz, CDCl_3_) δ 210.6 (C-1), 42.5 (C-2), 33.8 (C-3), 125.7 (C-4), 144.5 (C-5), 134.9 (C-6), 137.8 (C-7), 132.0 (C-8), 152.6 (C-9), 16.6 (C-11), 21.3 (C-12), 31.9 (C-13), 61.4 (C-14), 13.7 (C-15); ESI-MS *m/z* 219 [M + H]^+^.

**Pterosin Z (2)**. White amorphous powder; ^1^H-NMR (700 MHz, CDCl_3_) δ 7.10 (1H, s, H-4), 3.80 (2H, t, *J* = 7.7 Hz, H-14), 3.05 (2H, t, *J* = 7.7 Hz, H-13), 2.88 (2H, s, H-3), 2.71 (3H, s, H-15), 2.46 (3H, s, H-12), 1.22 (6H, s, H-10, 11); ^13^C-NMR (175 MHz, CDCl_3_) δ 212.3 (C-1), 45.6 (C-2), 44.7 (C-3), 125.9 (C-4), 144.4 (C-5), 134.8 (C-6), 138.4 (C-7), 131.1 (C-8), 151.4 (C-9), 25.6 (C-10, 11), 21.4 (C-12), 31.8 (C-13), 61.7 (C-14), 13.7 (C-15); ESI-MS *m/z* 233 [M + H]^+^.

**(2*S*)-pterosin P (3)**. White amorphous powder; ^1^H-NMR (700 MHz, CD_3_OD) δ 7.42 (1H, s, H-4), 4.75 (2H, s, H-12), 3.65 (2H, t, *J* = 7.7 Hz, H-14), 3.32 (1H, m, H_a_-3), 2.99 (1H, t, *J* = 7.7 Hz, H-13), 2.66 (1H, m, H-2), 2.65 (3H, s, H-15), 2.63 (1H, m, H_b_-3), 1.24 (3H, d, J = 7.0, H-11); ^13^C-NMR (175 MHz, CD_3_OD) δ 212.8 (C-1), 44.1 (C-2), 35.1 (C-3), 124.5 (C-4), 148.6 (C-5), 135.9 (C-6), 139.1 (C-7), 134.0 (C-8), 154.6 (C-9), 17.0 (C-11), 63.7 (C-12), 32.1 (C-13), 62.2 (C-14), 13.8 (C-15); ESI-MS *m/z* 235 [M + H]^+^.

**(3*S*)-pterosin D (4)**. White amorphous powder; ^1^H-NMR (700 MHz, CD_3_OD) δ 7.36 (1H, s, H-4), 4.74 (1H, s, H-3), 3.62 (2H, t, *J* = 7.7 Hz, H-14), 3.01 (2H, t, *J* = 7.7 Hz, H-13), 2.64 (3H, s, H-15), 2.48 (3H, s, H-12), 1.18 (3H, s, H-10), 1.03 (3H, s, H-11); ^13^C-NMR (175 MHz, CD_3_OD) δ 211.8 (C-1), 52.6 (C-2), 77.7 (C-3), 126.3 (C-4), 146.5 (C-5), 138.5^*^ (C-6), 138.6^*^ (C-7), 131.3 (C-8), 153.9 (C-9), 23.6 (C-10), 20.9 (C-11), 21.6 (C-12), 33.2 (C-13), 61.8 (C-14), 14.3 (C-15), ^*^interchangeable signals; ESI-MS *m/z* 249 [M + H]^+^.

**(2*S*)-pterosin A (5)**. White amorphous powder; ^1^H-NMR (700 MHz, CD_3_OD) δ 7.16 (1H, s, H-4), 3.70 (1H, d, *J* = 10.5 Hz, H_a_-11), 3.60 (2H, t, *J* = 7.7 Hz, H-14), 3.45 (1H, d, *J* = 10.5, H_b_-11), 3.23 (1H, d, *J* = 16.8 Hz, H_a_-3), 2.98 (2H, t, *J* = 7.7 Hz, H-13), 2.71 (1H, d, *J* = 16.8 Hz, H_b_-3), 2.64 (3H, s, H-15), 2.43 (3H, s, H-12), 1.08 (3H, s, H-10); ^13^C-NMR (175 MHz, CD_3_OD) δ 213.0 (C-1), 52.8 (C-2), 37.7 (C-3), 127.1 (C-4), 146.3 (C-5), 136.5 (C-6), 138.9 (C-7), 133.3 (C-8), 154.2 (C-9), 21.4 (C-10), 68.4 (C-11), 21.5 (C-12), 33.0 (C-13), 61.9 (C-14), 14.1 (C-15); ESI-MS *m/z* 249 [M + H]^+^, 271 [M + Na]^+^.

**(2*S*,3*R*)-pterosin C (6)**. White amorphous powder; ^1^H-NMR (700 MHz, CD_3_OD) δ 7.37 (1H, s, H-4), 5.15 (1H, d, *J* = 6.3 Hz, H-3), 3.61 (2H, t, *J* = 7.7 Hz, H-14), 3.01 (2H, t, *J* = 7.7 Hz, H-13), 2.76 (1H, t-like, *J* = 7.7 Hz, H-2), 2.65 (3H, s, H-15), 2.48 (3H, s, H-12), 1.17 (3H, *J* = 7.7 Hz, H-11); ^13^C-NMR (175 MHz, CD_3_OD) δ 210.5 (C-1), 49.8 (C-2), 70.4 (C-3), 126.9 (C-4), 146.4 (C-5), 138.7 (C-6), 138.2 (C-7), 132.4 (C-8), 155.3 (C-9), 10.9 (C-11), 21.5 (C-12), 33.2 (C-13), 61.8 (C-14), 14.2 (C-15); ESI-MS *m/z* 235 [M + H]^+^.

**(2*R*,3*R*)-pterosin C (7)**. White amorphous powder; [α]_D_^25^ ‒10.3 (c = 0.1, MeOH); ^1^H-NMR (700 MHz, CD_3_OD) δ 7.36 (1H, s, H-4), 4.69 (1H, d, *J* = 4.2 Hz, H-3), 3.61 (2H, t, *J* = 7.7 Hz, H-14), 3.01 (2H, t, *J* = 7.7 Hz, H-13), 2.44 (1H, dd, *J* = 7.7, 4.2 Hz, H-2), 2.64 (3H, s, H-15), 2.48 (3H, s, H-12), 1.30 (3H, *J* = 7.7 Hz, H-11); ^13^C-NMR (175 MHz, CD_3_OD) δ 207.8 (C-1), 54.9 (C-2), 76.0 (C-3), 125.8 (C-4), 146.4 (C-5), 138.4 (C-6), 138.0 (C-7), 132.6 (C-8), 154.7 (C-9), 13.4 (C-11), 21.6 (C-12), 33.2 (C-13), 61.8 (C-14), 14.2 (C-15); ESI-MS *m/z* 235 [M + H]^+^.

**(2*R*)-pteroside B (8)**. White amorphous powder; ^1^H-NMR (700 MHz, CD_3_OD) δ 7.13 (1H, s, H-4), 4.31 (1H, d, *J* = 7.7 Hz, H-1'), 3.90 (1H, dd, *J* = 16.8, 7.7 Hz, H_a_-14), 3.85 (1H, d, *J* = 11.9, H_a_-6'), 3.66 (1H, dd, *J* = 11.9, 4.9 Hz, H_b_-6'), 3.64 (1H, m, H_b_-14), 3.35 (1H, t, *J* = 9.1 Hz, H-3'), 3.30 (1H, t, *J* = 9.1 Hz, H-4'), 3.25 (1H, m, H_a_-3), 3.20 (1H, t, *J* = 9.1 Hz, H-2'), 3.09 (2H, t, *J* = 7.7 Hz, H-13), 2.64 (3H, s, H-15), 2.61 (1H, m, H-2), 2.56 (1H, brd, *J* = 16.8 Hz, H_b_-3), 2.44 (3H, s, H-12), 1.22 (3H, d, *J* = 7.0 Hz, H-11); ^13^C-NMR (175 MHz, CD_3_OD) δ 212.7 (C-1), 43.9 (C-2), 34.9 (C-3), 127.0 (C-4), 146.4 (C-5), 136.4 (C-6), 139.0 (C-7), 133.0 (C-8), 154.6 (C-9), 17.0 (C-11), 21.5 (C-12), 30.2 (C-13), 69.3 (C-14), 14.0 (C-15), 104.6 (C-1'), 75.3 (C-2'), 78.3 (C-3'), 71.7 (C-4'), 78.1 (C-5'), 62.8 (C-6'); ESI-MS *m/z* 403 [M + Na]^+^.

**Pteroside Z (9)**. White amorphous powder; ^1^H-NMR (700 MHz, CD_3_OD) δ 7.13 (1H, s, H-4), 4.32 (1H, d, *J* = 7.7 Hz, H-1'), 3.90 (1H, dd, *J* = 16.8, 7.7 Hz, H_a_-14), 3.85 (1H, d, *J* = 11.9, H_a_-6'), 3.66 (1H, dd, *J* = 11.9, 4.9 Hz, H_b_-6'), 3.64 (1H, m, H_b_-14), 3.35 (1H, t, *J* = 9.1 Hz, H-3'), 3.30 (1H, t, *J* = 9.1 Hz, H-4'), 3.20 (1H, t, *J* = 9.1 Hz, H-2'), 3.11 (2H, t, *J* = 7.7 Hz, H-13), 2.86 (2H, s, H-3), 2.65 (3H, s, H-15), 2.45 (3H, s, H-12), 1.52 (6H, s, H-10, 11); ^13^C-NMR (175 MHz, CD_3_OD) δ 214.7 (C-1), 46.8 (C-2), 42.7 (C-3), 127.1 (C-4), 146.6 (C-5), 136.5 (C-6), 139.4 (C-7), 132.0 (C-8), 153.2 (C-9), 26.0 (C-10, 11), 21.5 (C-12), 30.2 (C-13), 69.3 (C-14), 14.0 (C-15), 104.6 (C-1'), 75.3 (C-2'), 78.3 (C-3'), 71.7 (C-4'), 78.1 (C-5'), 62.8 (C-6'); ESI-MS *m/z* 417 [M + Na]^+^.

**(2*S*)-pteroside A (10)**. White amorphous powder; [α]_D_^25^ ‒46.3 (c = 0.9, MeOH); ^1^H-NMR (700 MHz, CD_3_OD) δ 7.12 (1H, s, H-4), 4.31 (1H, d, *J* = 7.7 Hz, H-1'), 3.91 (1H, dd, *J* = 16.8, 7.7 Hz, H_a_-14), 3.84 (1H, d, *J* = 11.9 Hz, H_a_-6'), 3.70 (1H, d, *J* = 10.5 Hz, H_a_-11), 3.66 (1H, dd, *J* = 11.9, 4.9 Hz, H_b_-6'), 3.64 (1H, m, H_b_-14), 3.46 (1H, d, *J* = 10.5, H_b_-11), 3.35 (1H, t, *J* = 9.1 Hz, H-3'), 3.30 (1H, t, *J* = 9.1 Hz, H-4'), 3.23 (1H, d, *J* = 16.8 Hz, H_a_-3), 3.19 (1H, t, *J* = 9.1 Hz, H-2'), 3.10 (2H, t, *J* = 7.7 Hz, H-13), 2.71 (1H, d, *J* = 16.8 Hz, H_b_-3), 2.66 (3H, s, H-15), 2.45 (3H, s, H-12), 1.08 (3H, s, H-10); ^13^C-NMR (175 MHz, CD_3_OD) δ 213.0 (C-1), 52.8 (C-2), 37.6 (C-3), 127.1 (C-4), 146.5 (C-5), 136.3 (C-6), 139.1 (C-7), 133.2 (C-8), 154.3 (C-9), 21.4 (C-10), 68.4 (C-11), 21.5 (C-12), 30.2 (C-13), 69.3 (C-14), 14.1 (C-15), 104.6 (C-1'), 75.3 (C-2'), 78.3 (C-3'), 71.7 (C-4'), 78.1 (C-5'), 62.8 (C-6'); ESI-MS *m/z* 411 [M + H]^+^.

**(2*S*)-pteroside A_2_ (11)**. White amorphous powder; [α] _D_^25^ ‒43.5 (c = 0.3, MeOH); 1H-NMR (700 MHz, CD_3_OD) δ 7.16 (1H, s, H-4), 4.20 (1H, d, *J* = 7.7 Hz, H-1'), 4.12 (1H, d, *J* = 9.1 Hz, Ha-11), 3.76 (1H, d, *J* = 11.9 Hz, Ha-6'), 3.60 (3H, overlapped, H-14, H_b_-6'), 3.47 (1H, d, *J* = 9.1, H_b_-11), 3.42 (1H, d, *J* = 16.8 Hz, H_a_-3), 3.29 (1H, t, *J* = 9.1 Hz, H-3'), 3.21 (2H, overlapped H-4', 5'), 3.03 (1H, t, *J* = 9.1 Hz, H-2'), 2.98 (2H, t, *J* = 7.7 Hz, H-13), 2.72 (1H, d, *J* = 16.8 Hz, H_b_-3), 2.64 (3H, s, H-15), 2.44 (3H, s, H-12), 1.12 (3H, s, H-10); ^13^C-NMR (175 MHz, CD_3_OD) δ 212.6 (C-1), 51.5 (C-2), 37.7 (C-3), 127.2 (C-4), 146.5 (C-5), 136.6 (C-6), 139.1 (C-7), 132.7 (C-8), 154.3 (C-9), 22.2 (C-10), 75.2 (C-11), 21.5 (C-12), 33.0 (C-13), 61.8 (C-14), 14.1 (C-15), 104.9 (C-1'), 75.0 (C-2'), 78.3 (C-3'), 71.7 (C-4'), 78.0 (C-5'), 62.8 (C-6'); ESI-MS *m/z* 433 [M + Na]^+^.

**(2*S*,3*R*)-pteroside C (12)**. White amorphous powder; [α] _D_^25^ ‒29.3 (c = 0.8, MeOH); ^1^H-NMR (700 MHz, CD_3_OD) δ 7.36 (1H, s, H-4), 5.14 (1H, d, *J* = 6.3 Hz, H-3), 4.31 (1H, d, *J* = 7.7 Hz, H-1'), 3.90 (1H, dd, *J* = 16.8, 7.7 Hz, H_a_-14), 3.85 (1H, d, *J* = 11.9 Hz, H_a_-6'), 3.66 (1H, d, *J* = 11.9 Hz, H_b_-6'), 3.65 (1H, m, H_b_-14), 3.35 (1H, t, *J* = 9.1 Hz, H-3'), 3.19 (1H, t, *J* = 9.1 Hz, H-2'), 3.12 (2H, t, *J* = 7.7 Hz, H-13), 2.75 (1H, t-like, *J* = 7.7 Hz, H-2), 2.66 (3H, s, H-15), 2.49 (3H, s, H-12), 1.17 (3H, *J* = 7.0 Hz, H-11); ^13^C-NMR (175 MHz, CD_3_OD) δ 210.5 (C-1), 49.8 (C-2), 70.4 (C-3), 126.8 (C-4), 146.6 (C-5), 138.4 (C-6), 138.4 (C-7), 132.3 (C-8), 155.4 (C-9), 10.9 (C-11), 21.6 (C-12), 30.4 (C-13), 69.2 (C-14), 14.2 (C-15), 104.6 (C-1'), 75.6 (C-2'), 78.3 (C-3'), 71.7 (C-4'), 78.1 (C-5'), 62.8 (C-6'); ESI-MS *m/z* 419 [M + Na]^+^.

**(2*R*,3*R*)-pteroside C (13)**. White amorphous powder; [α] _D_^25^ ‒48.5 (c = 0.9, MeOH); ^1^H-NMR (700 MHz, CD_3_OD) δ 7.36 (1H, s, H-4), 4.38 (1H, d, *J* = 3.5 Hz, H-3), 4.31 (1H, d, *J* = 7.7 Hz, H-1'), 3.90 (1H, dd, *J* = 16.8, 7.7 Hz, H_a_-14), 3.85 (1H, d, *J* = 11.9 Hz, H_a_-6'), 3.66 (1H, d, *J* = 11.9 Hz, H_b_-6'), 3.65 (1H, m, H_b_-14), 3.35 (1H, t, *J* = 9.1 Hz, H-3'), 3.19 (1H, t, *J* = 9.1 Hz, H-2'), 3.12 (2H, t, *J* = 7.7 Hz, H-13), 2.44 (1H, dd, *J* = 7.0, 4.2 Hz, H-2), 2.66 (3H, s, H-15), 2.49 (3H, s, H-12), 1.29 (3H, *J* = 7.0 Hz, H-11); ^13^C-NMR (175 MHz, CD_3_OD) δ 210.7 (C-1), 54.8 (C-2), 76.0 (C-3), 125.8 (C-4), 146.6 (C-5), 138.2 (C-6), 138.1 (C-7), 132.5 (C-8), 154.8 (C-9), 13.4 (C-11), 21.6 (C-12), 30.4 (C-13), 69.2 (C-14), 14.2 (C-15), 104.6 (C-1'), 75.3 (C-2'), 78.3 (C-3'), 71.7 (C-4'), 78.1 (C-5'), 62.8 (C-6'); ESI-MS *m/z* 419 [M + Na]^+^.

**(3*S*)-pteroside D (14)**. White amorphous powder; [α] _D_^25^ ‒43.6 (c = 0.8, MeOH); ^1^H-NMR (700 MHz, CD_3_OD) δ 7.35 (1H, s, H-4), 4.74 (1H, s, H-3), 4.31 (1H, d, *J* = 7.7 Hz, H-1'), 3.91 (1H, dd, *J* = 16.8, 7.7 Hz, H_a_-14), 3.85 (1H, d, *J* = 11.9 Hz, H_a_-6'), 3.66 (1H, d, *J* = 11.9 Hz, H_b_-6'), 3.65 (1H, m, H_b_-14), 3.35 (1H, t, *J* = 9.1 Hz, H-3'), 3.19 (1H, t, *J* = 9.1 Hz, H-2'), 3.13 (2H, t, *J* = 7.7 Hz, H-13), 2.65 (3H, s, H-15), 2.49 (3H, s, H-12), 1.18 (3H, s, H-11), 1.03 (3H, s, H-10); ^13^C-NMR (175 MHz, CD_3_OD) δ 211.8 (C-1), 52.6 (C-2), 77.7 (C-3), 126.3 (C-4), 146.7 (C-5), 138.3 (C-6), 138.7 (C-7), 131.3 (C-8), 154.0 (C-9), 20.9 (C-10), 23.6 (C-11), 21.6 (C-12), 30.4 (C-13), 69.2 (C-14), 14.3 (C-15), 104.6 (C-1'), 75.3 (C-2'), 78.3 (C-3'), 71.7 (C-4'), 78.1 (C-5'), 62.8 (C-6'); ESI-MS *m/z* 433 [M + Na]^+^.

**(2*S*)-pteroside P (15)**. White amorphous powder; [α] _D_^25^ ‒41.5 (c = 0.1, MeOH); ^1^H-NMR (700 MHz, CD_3_OD) δ 7.42 (1H, s, H-4), 4.78 (2H, s, H-12), 4.29 (1H, d, *J* = 7.7 Hz, H-1'), 3.34 (1H, t, *J* = 9.1 Hz, H-3'), 3.18 (1H, t, *J* = 9.1 Hz, H-2'), 3.11 (2H, t, *J* = 7.7 Hz, H-14), 2.66 (3H, s, H-15), 2.65 (1H, m, H-2), 2.63 (1H, d, *J* = 14.0, 2.8 Hz, H-13), 1.24 (3H, d, J = 7.0, H-11); ^13^C-NMR (175 MHz, CD_3_OD) δ 212.8 (C-1), 44.1 (C-2), 35.1 (C-3), 124.4 (C-4), 148.8 (C-5), 135.6 (C-6), 139.2 (C-7), 133.9 (C-8), 154.6 (C-9), 17.0 (C-11), 63.7 (C-12), 29.4 (C-13), 70.0 (C-14), 14.3 (C-15), 104.6 (C-1'), 75.3 (C-2'), 78.3 (C-3'), 71.7 (C-4'), 78.2 (C-5'), 62.8 (C-6'); ESI-MS *m/z* 419 [M + Na]^+^.

**Supplementary Information 2**

**C**

**B**

**A**

**D**

**E**

**F**

Dixon plots for BACE1 inhibition by pterosin derivatives. (2*S*,3*R*)-Pterosin C (A), (2*R*,3*R*)-pterosin C (B), and (2*R*)-pterosin B (C) were tested in the presence of substrate concentrations of 150 nM (●), 250 nM (○), and 750 nM (▼). Lineweaver-Burk plots for BACE1 inhibition were generated with inhibitor concentrations of 0 µM (■), 2.5 µM (Δ), 12.5 µM (▼), 62.5 µM (○), and 125 µM (●) for (2*S*,3*R*)-pterosin C (D), (2*R*,3*R*)-pterosin C (E), and (2*R*)-pterosin B (F), respectively.

**A**

**B**

**C**

**D**

**E**

**F**

Dixon plots for BACE1 inhibition by pteroside derivatives. (2*R*)-pteroside B (A), (2*R*,3*R*)-pteroside C (B), and (3*S*)-pteroside D (C) were tested in the presence of substrate concentrations of 150 nM (●), 250 nM (○), and 750 nM (▼). Lineweaver-Burk plots for BACE1 inhibition were generated with inhibitor concentrations of 0 µM (■), 2.5 µM (Δ), 12.5 µM (▼), 62.5 µM (○), and 125 µM (●) for (2*R*)-pteroside B (D); 0 µM (■), 0.5 µM (Δ), 2.5 µM (▼), 12.5 µM (○), and 62.5 µM (●) for (2*R*,3*R*)-pteroside C (E) and (3*S*)-pteroside D (F).

**Supplementary Information 2**

**A**

**B**

**D**

**C**

**E**

**F**

Dixon plots for AChE inhibition by pterosin derivatives. (2*S*,3*R*)-Pterosin C (A), (2*R*,3*R*)-pterosin C (B), and (2*R*)-pterosin B (C) were tested in the presence of substrate concentrations of 0.1 mM (●), 0.3 mM (○), and 0.6 mM (▼). Lineweaver-Burk plots for AChE inhibition were generated with inhibitor concentrations of 0 µM (Δ), 4 µM (▼), 20 µM (○), and 100 µM (●) for (2*S*,3*R*)-pterosin C (D) and (2*R*,3*R*)-pterosin C (E); and 0 µM (Δ), 2 µM (▼), 10 µM (○), and 50 µM (●) for (2*R*)-pterosin B (F).

**A**

**B**

**C**

**D**

**E**

**F**

Dixon plots for AChE inhibition by pteroside derivatives. (2*R*)-pteroside B (A), (2*R*,3*R*)-pteroside C (B), and (3*S*)-pteroside D (C) were tested in the presence of substrate concentrations of 0.1 mM (●), 0.3 mM (○), and 0.6 mM (▼). Lineweaver-Burk plots for AChE inhibition were generated with inhibitor concentrations of 0 µM (Δ), 0.8 µM (▼), 4 µM (○), and 20 µM (●) for (2*R*)-pteroside B (D), (2*R*,3*R*)-pteroside C (E), and (3*S*)-pteroside D (F).

**Supplementary Information 2**

**A**

**B**

**C**

**D**

**E**

**F**

Dixon plots for BChE inhibition by pterosin derivatives. (2*S*,3*R*)-Pterosin C (A), (2*R*,3*R*)-pterosin C (B), and (2*R*)-pterosin B (C) were tested in the presence of substrate concentrations of 0.2 mM (●), 0.4 mM (○), and 0.8 mM (▼). Lineweaver-Burk plots for BChE inhibition were generated with inhibitor concentrations of 0 µM (Δ), 50 µM (▼), 100 µM (○), and 150 µM (●) for (2*S*,3*R*)-pterosin C (D); 0 µM (Δ), 0.8 µM (▼), 4 µM (○), and 20 µM (●) for (2*R*,3*R*)-pterosin C (E); and 0 µM (Δ), 10 µM (▼), 50 µM (○), and 100 µM (●) for (2*R*)-pterosin B (F).

**A**

**B**

**C**

**D**

**E**

**F**

Dixon plots for BChE inhibition by pteroside derivatives. (2*R*)-pteroside B (A), (2*R*,3*R*)-pteroside C (B), and (3*S*)-pteroside D (C) were tested in the presence of substrate concentrations of 0.2 mM (●), 0.4 mM (○), and 0.8 mM (▼). Lineweaver-Burk plots for BChE inhibition were generated with inhibitor concentrations of 0 µM (Δ), 4 µM (▼), 20 µM (○), and 100 µM (●) for (2*R*)-pteroside B (D); 0 µM (Δ), 0.8 µM (▼), 4 µM (○), and 20 µM (●) for (2*R*,3*R*)-pteroside C (E) and (3*S*)-pteroside D (F).

**Supplementary Information 3**


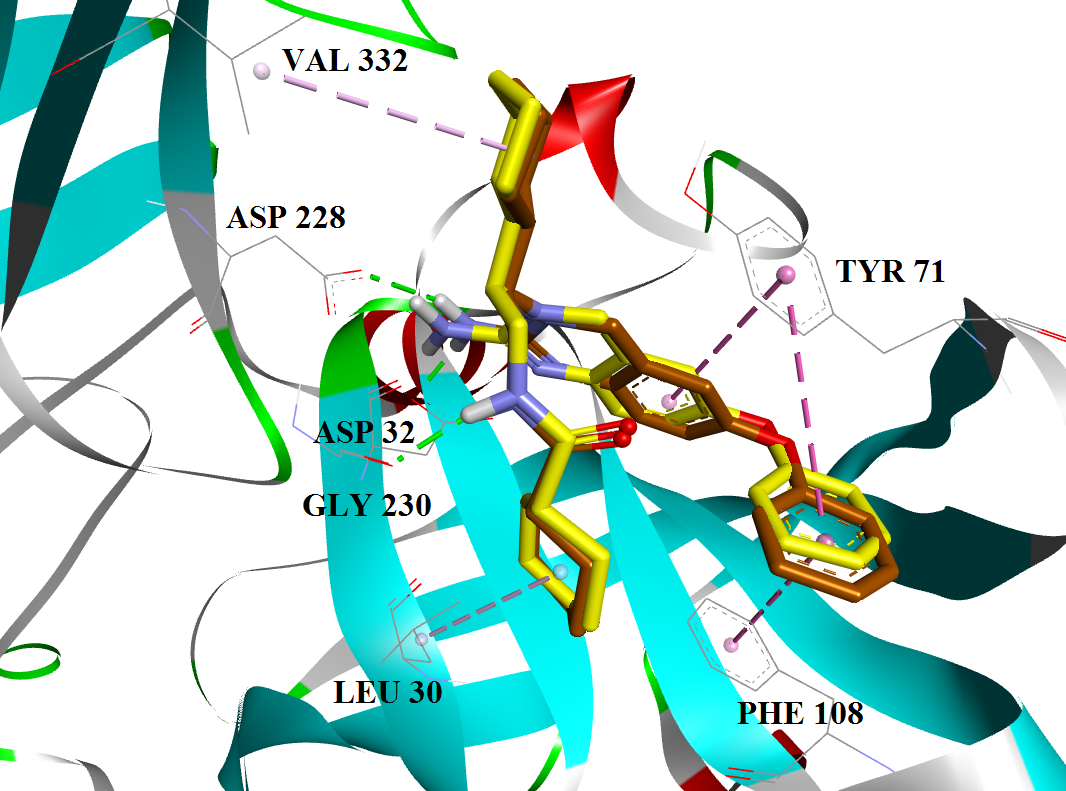


Conformational comparison of QUD from the crystal structure (yellow), PDB 2WJO and from docking results (brown). BACE1 active site residues are represented by line model and ligand is displayed as stick model. Colors of the dotted lines explain the types of various interactions: hydrogen bonding interactions (green) and hydrophobic interactions (pink).

**Supplementary Information 3**


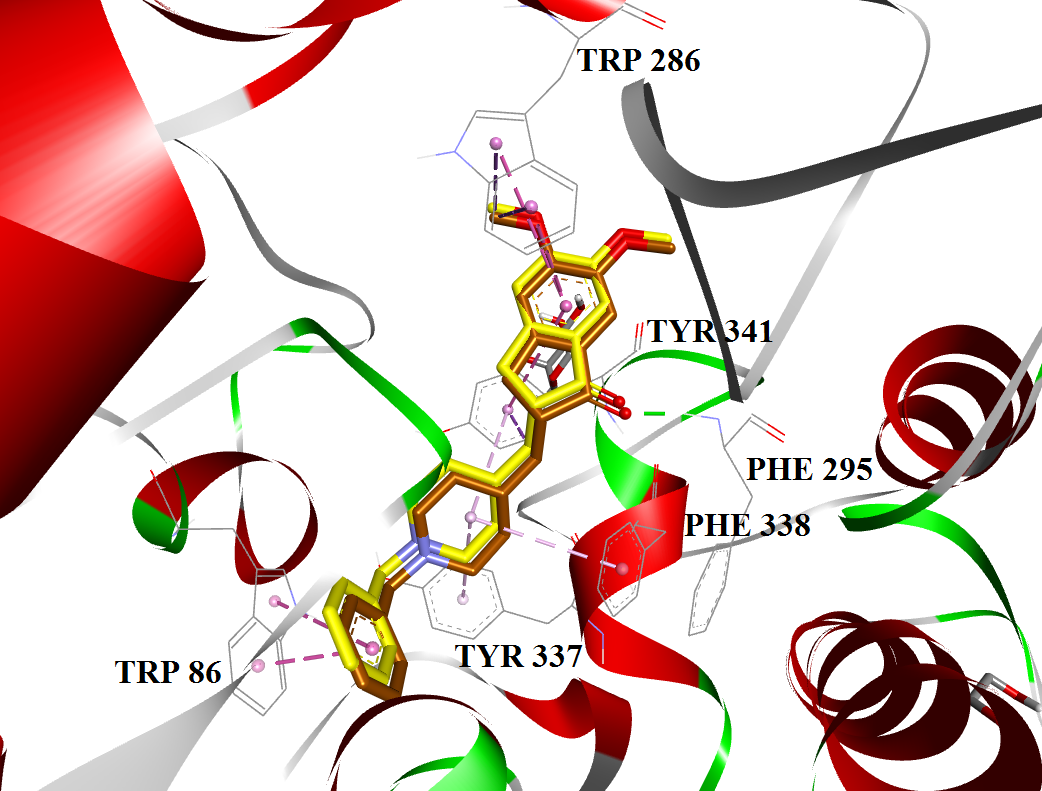


Conformational comparison of E2020 (donepezil) from the crystal structure (yellow), PDB 4EY7 and from docking results (brown). AChE active site residues are represented by line model and ligand is displayed as stick model. Colors of the dotted lines explain the types of various interactions: hydrogen bonding interactions (green), hydrophobic interactions (pink) and π-sigma interactions (purple).

**Supplementary Information 3**


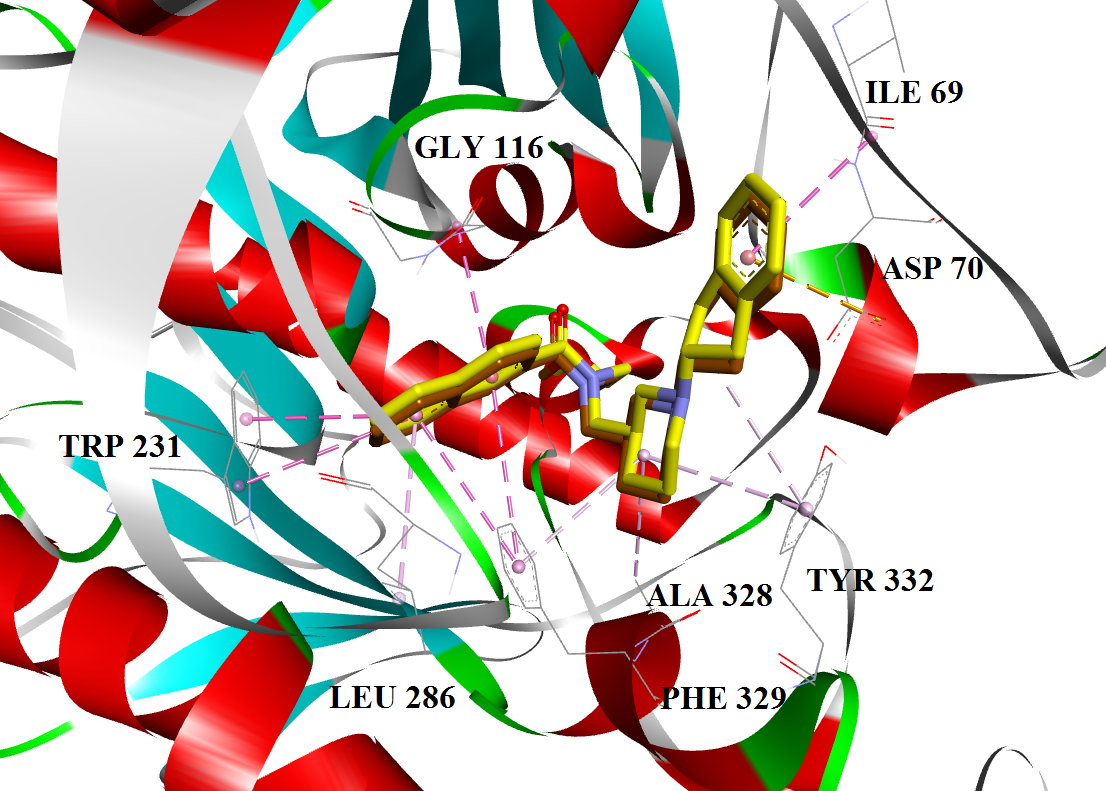


Crystal and docked conformations of 3F9. Conformational comparison of 3F9 from the crystal structure (yellow), PDB 4TPK and from docking results (brown). BChE active site residues are represented by line model and ligand is displayed as stick model. Colors of the dotted lines explain the types of various interactions: hydrogen bonding interactions (green), hydrophobic interactions (pink) and π-anion interactions (golden).

**Supplementary Information 4**

**BBB permeability of verapamil (control)**

*P*_e_ (10^-6^ cm/s): 34.6 ± 3.9, log *P*_e_: -4.46


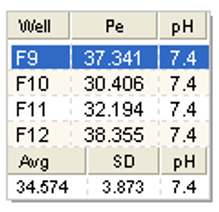

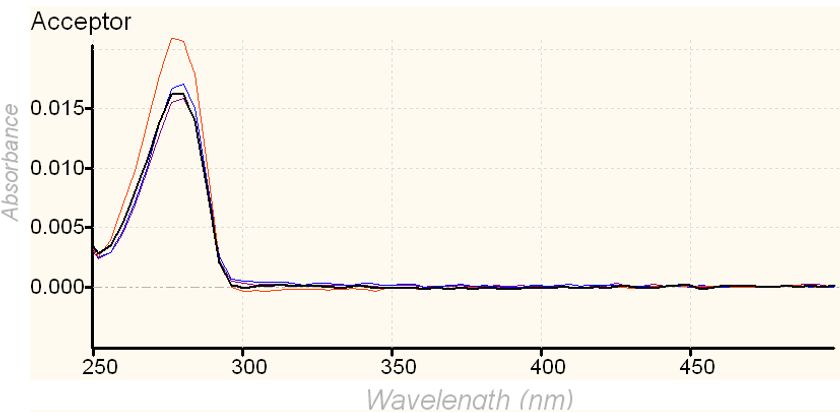


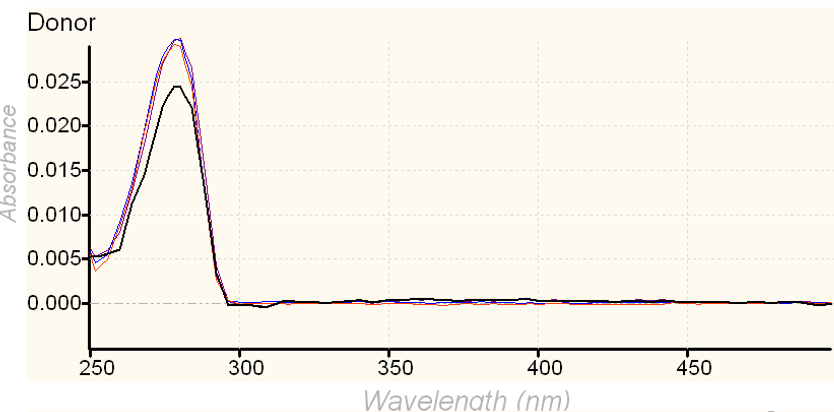


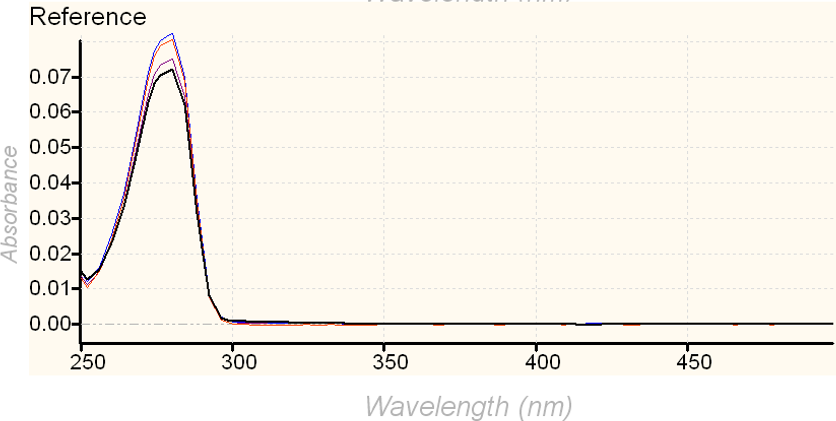


The box at the top right presents the *P*_e_ values obtained from quadruple PAMPA-BBB for verapamil. PAMPA-BBB data indicated in blue in the box is illustrated graphically.

**BBB permeability of pterosin derivatives**

**(2*R*)-Pterosin B**


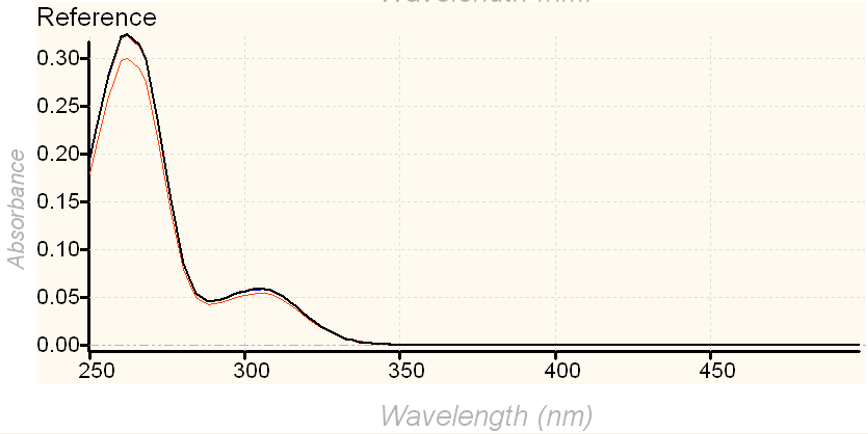

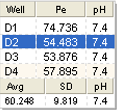

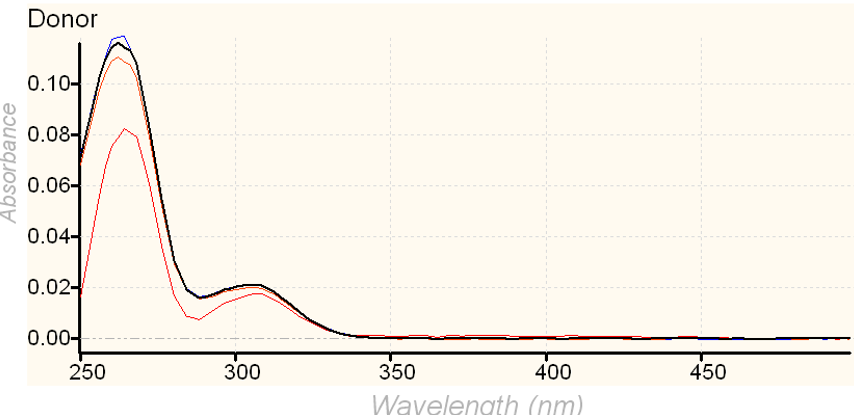

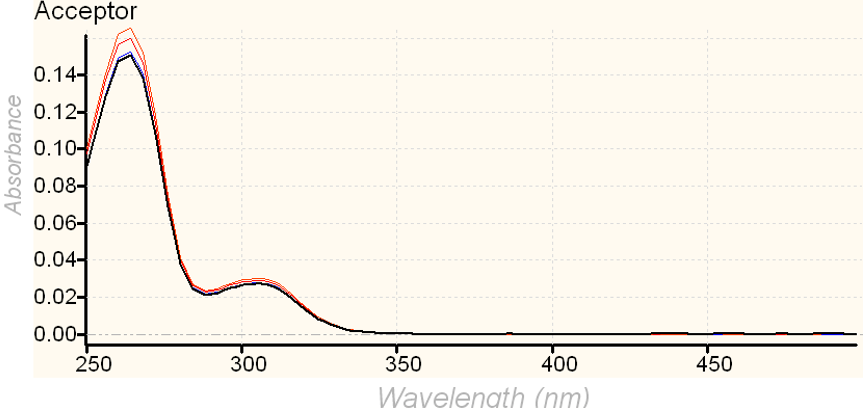
*P*_e_ (10^-6^ cm/s): 60.3 ± 9.8, log *P*_e_: -4.22

The box at the top right presents the *P*_e_ values obtained from quadruple PAMPA-BBB for (2*R*)-pterosin B. PAMPA-BBB data indicated in blue in the box is illustrated graphically.

**(2*S*)-Pterosin P**


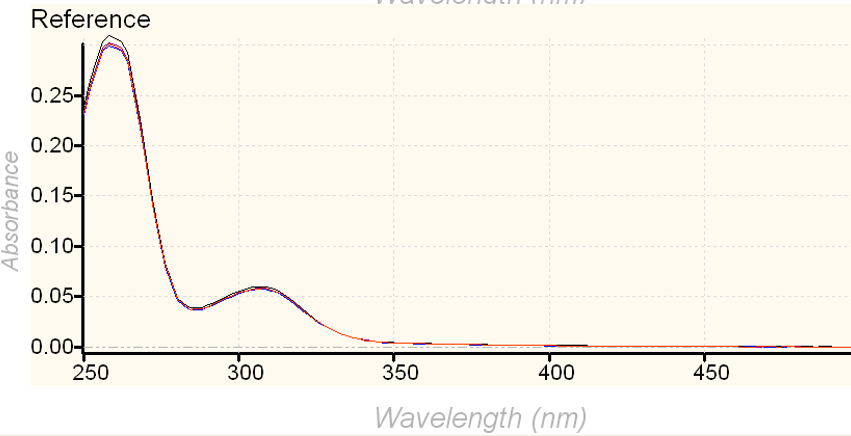

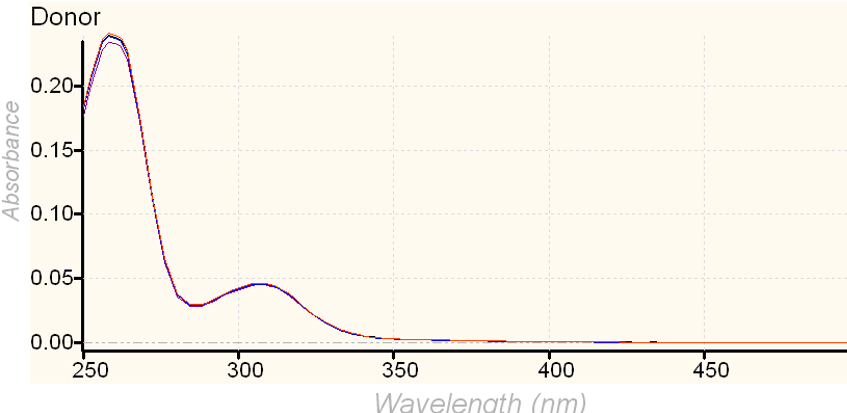

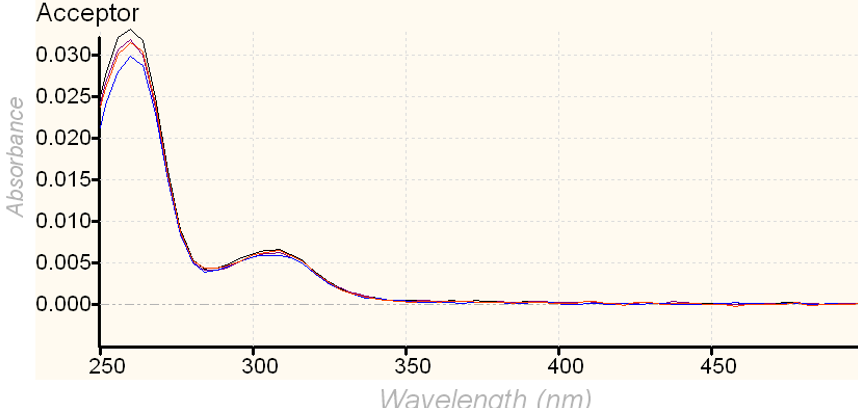

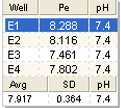
*P*_e_ (10^-6^ cm/s): 7.92 ± 0.36, log *P*_e_: -5.10

The box at the top right presents the *P*_e_ values obtained from quadruple PAMPA-BBB for (2*S*)-pterosin P. PAMPA-BBB data indicated in blue in the box is illustrated graphically.

**(2*S*)-Pterosin A**


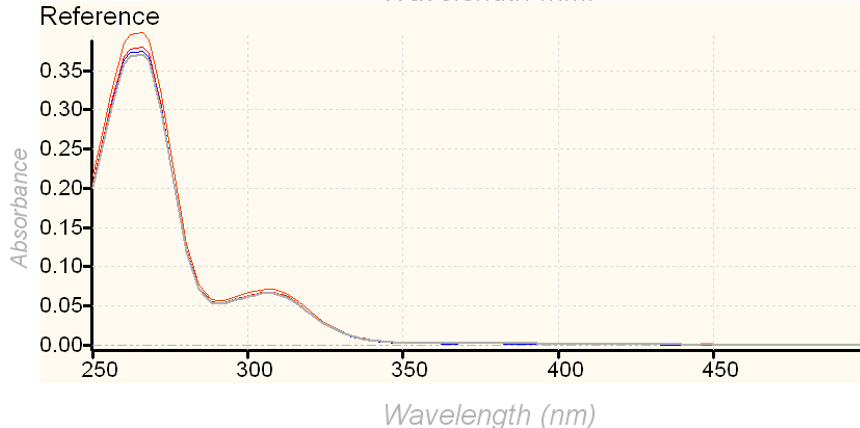

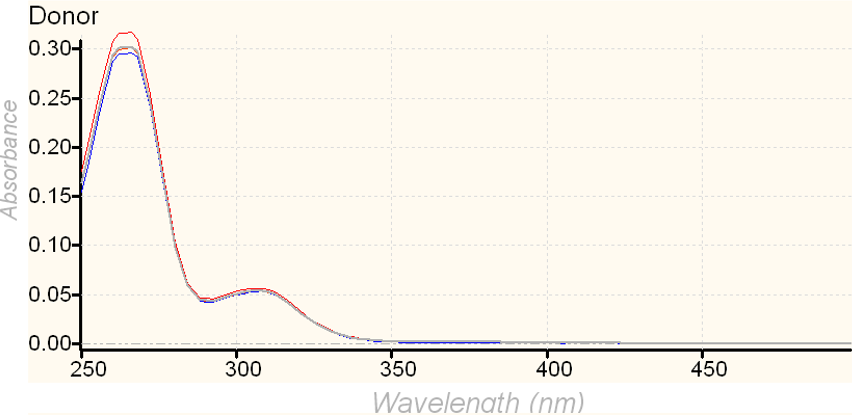

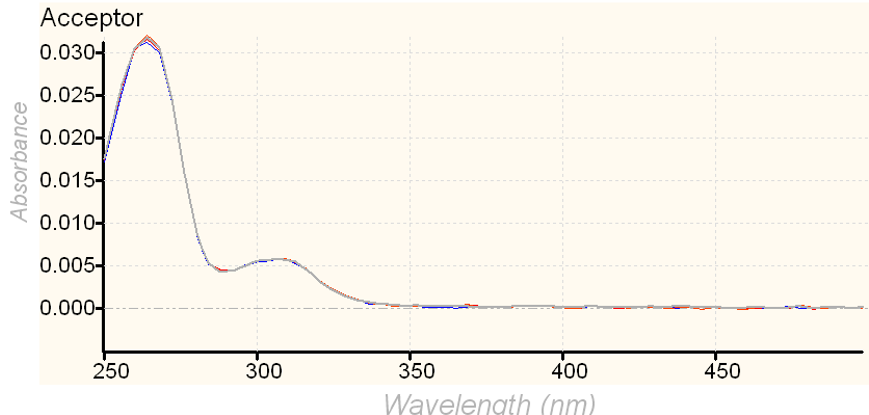

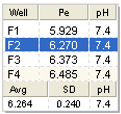
*P*_e_ (10^-6^ cm/s): 6.26 ± 0.24, log *P*_e_: -5.20

The box at the top right presents the *P*_e_ values obtained from quadruple PAMPA-BBB for (2*S*)-pterosin A. PAMPA-BBB data indicated in blue in the box is illustrated graphically.

**(2*S*,3*R*)-Pterosin C**


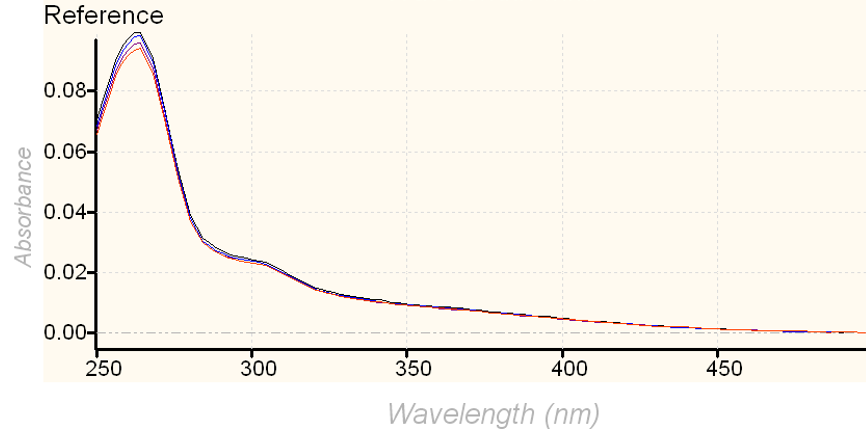

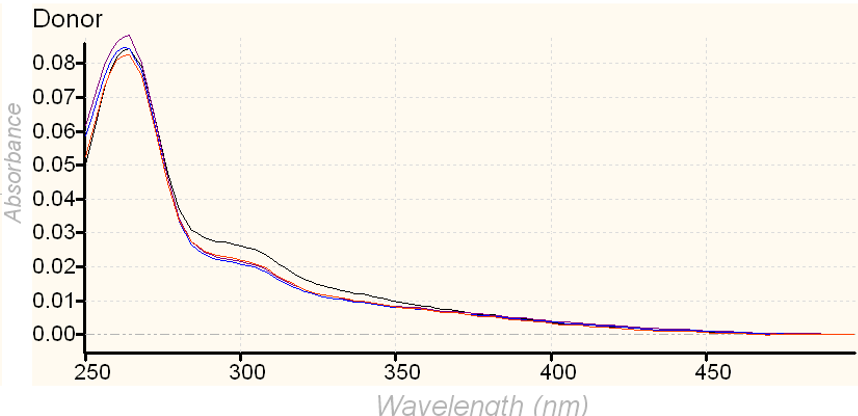

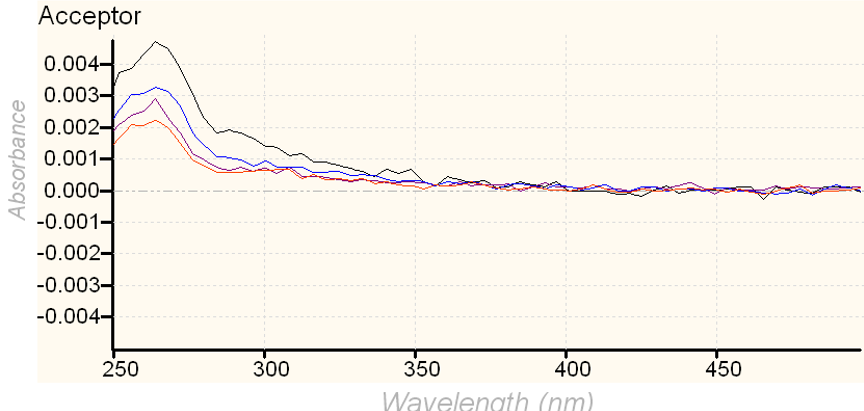

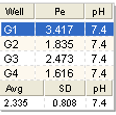
*P*_e_ (10^-6^ cm/s): 2.34 ± 0.81, log *P*_e_: -5.65

The box at the top right presents the *P*_e_ values obtained from quadruple PAMPA-BBB for (2*S*,3*R*)-pterosin C. PAMPA-BBB data indicated in blue in the box is illustrated graphically.

**(2*R*,3*R*)-Pterosin C**


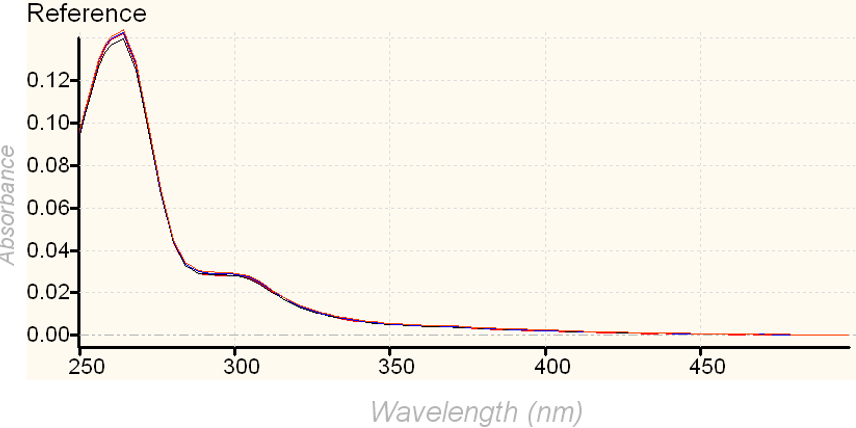

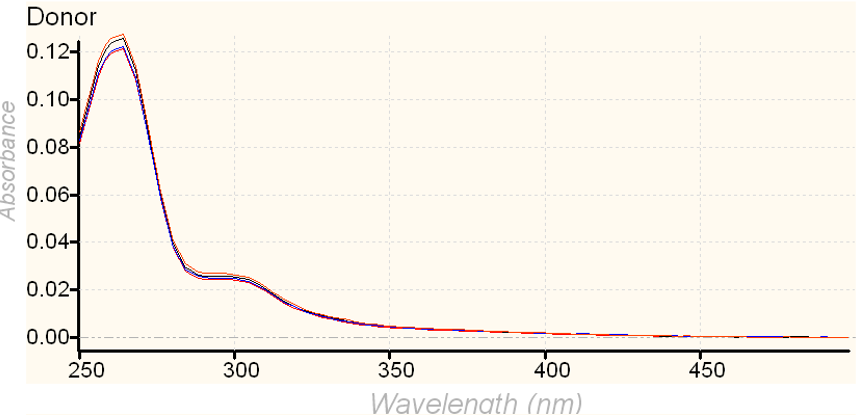

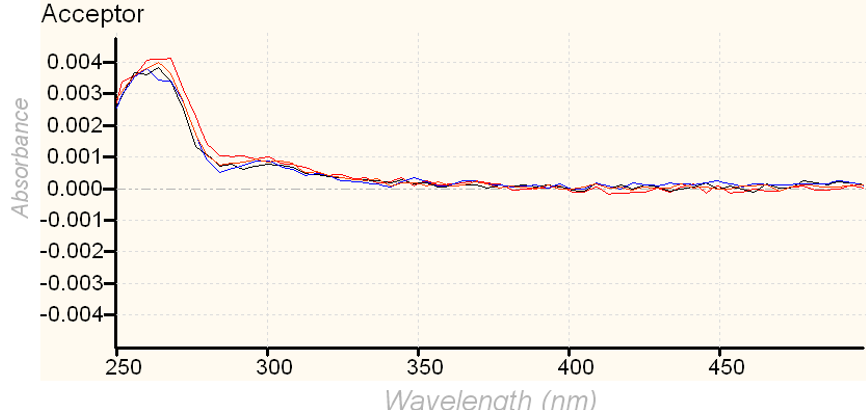

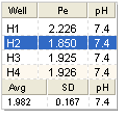
*P*_e_ (10^-6^ cm/s): 1.98 ± 0.17, log *P*_e_: -5.70

The box at the top right presents the *P*_e_ values obtained from quadruple PAMPA-BBB for (2*R*,3*R*)-pterosin C. PAMPA-BBB data indicated in blue in the box is illustrated graphically.

**Supplementary Information 5**

**Cytotoxicity of Pterosin Derivatives**

| **Compounds** | **LD_50_ (µM) Based on MTT Assay** | | | |
| --- | --- | --- | --- | --- |
|  | **Normal cell** | | | **Cancer cell** |
|  | **SY-SY5Y** | **C6** | **NIH3T3** | **B16F10** |
| (2*S*)-Pterosin A | 1,210 ± 110 | >5,000 | >5,000 | 522 ± 25 |
| (2*R*)-Pterosin B | >5,000 | >5,000 | >5,000 | >5,000 |
| (2*S*,3*R*)-Pterosin C | 2,940 ± 260 | >5,000 | >5,000 | >5,000 |
| (2*R*,3*R*)-Pterosin C | 719 ± 25 | 694 ± 9.7 | >5,000 | >5,000 |
| (3*R*)-Pterosin D | >5,000 | >5,000 | >5,000 | >5,000 |
| (2*S*)-Pterosin P | >5,000 | >5,000 | >5,000 | >5,000 |
| Pterosin Z | 495 ± 24 | 1,850 ± 65 | 553 ± 37 | 618 ± 71 |
| (2*S*)-Pteroside A | >5,000 | >5,000 | >5,000 | >5,000 |
| (2*S*)-Pteroside A_2_ | 1,010 ± 60 | >5,000 | >5,000 | >5,000 |
| (2*R*)-Pteroside B | >5,000 | >5,000 | >5,000 | >5,000 |
| (2*S*,3*R*)-Pteroside C | 711 ± 35 | >5,000 | >5,000 | >5,000 |
| (2*R*,3*R*)-Pteroside C | >5,000 | >5,000 | >5,000 | >5,000 |
| (3*S*)-Pteroside D | 1,320 ± 86 | >5,000 | >5,000 | >5,000 |
| (2S)-Pteroside P | 1,090 ± 99 | >5,000 | >5,000 | >5,000 |
| Pteroside Z | 1,430 ± 210 | 2,130 ± 62 | >5,000 | >5,000 |

Values are mean ± S.D. obtained from three independent experiments.

SH-SY5Y, human neuroblastoma; C6, rat glial cell; NIH3T3, mouse embryo fibroblast; B16F10, mouse melanoma.
